# A cryo-ET survey of microtubules and intracellular compartments in mammalian axons

**DOI:** 10.1083/jcb.202103154

**Published:** 2021-12-08

**Authors:** Helen E. Foster, Camilla Ventura Santos, Andrew P. Carter

**Affiliations:** MRC Laboratory of Molecular Biology, Cambridge, UK

## Abstract

The neuronal axon is packed with cytoskeletal filaments, membranes, and organelles, many of which move between the cell body and axon tip. Here, we used cryo-electron tomography to survey the internal components of mammalian sensory axons. We determined the polarity of the axonal microtubules (MTs) by combining subtomogram classification and visual inspection, finding MT plus and minus ends are structurally similar. Subtomogram averaging of globular densities in the MT lumen suggests they have a defined structure, which is surprising given they likely contain the disordered protein MAP6. We found the endoplasmic reticulum in axons is tethered to MTs through multiple short linkers. We surveyed membrane-bound cargos and describe unexpected internal features such as granules and broken membranes. In addition, we detected proteinaceous compartments, including numerous virus-like capsid particles. Our observations outline novel features of axonal cargos and MTs, providing a platform for identification of their constituents.

## Teaser

Foster et al. use cryo-electron tomography to survey the mammalian axoplasm. Highlights include determination of microtubule polarity by classification, subtomogram averaging of microtubule inner proteins (MIPs), a description of tethers linking the ER to microtubules, and the observation of numerous virus-like capsids in the axoplasm.

## Introduction

Axons are slender processes that extend from the neuronal cell body to transmit electrical signals to neighboring cells. Their outer membrane sheath is filled with an axoplasm containing cytoskeletal filaments and many membrane-bound organelles (Hirokawa, 1982; Schnapp and Reese, 1982). Proper axonal functions require a constant supply of components to the synaptic termini and their recycling back to the cell body. This intracellular transport relies on the microtubule (MT) cytoskeleton, which lines and shapes the axon (Kapitein and Hoogenraad, 2015).

MTs are long, polar filaments that in axons are predominantly oriented with their plus ends toward the synapse and their minus ends toward the cell body (Baas et al., 1988; Heidemann et al., 1981). A proportion of these MTs are dynamic and grow from the plus end (Baas and Black, 1990; Stepanova et al., 2003). Cellular MTs can be stabilized through binding of MT-associated proteins (MAPs) or via posttranslational modifications (Janke and Magiera, 2020). Axonal MTs are highly stable and remain intact even after cold treatment (Brady et al., 1984; Song et al., 2013). They contain many globular luminal particles (Garvalov et al., 2006), also referred to as MT inner proteins (MIPs), which are thought to contain a cold-stability factor, MAP6 (Cuveillier et al., 2020). Although axonal MTs are well studied, a number of questions regarding how they grow and are stabilized remain unanswered.

MTs provide the tracks for motor proteins to transport intracellular cargos. Movement toward the axon tip is driven by kinesin motors. Their cargos include Golgi-derived vesicles containing neuropeptides (Barkus et al., 2008), transmembrane channels (Akin et al., 2019), and receptors (Zhao et al., 2011). The return of components to the cell body depends on cytoplasmic dynein-1, which is responsible for transporting endocytic vesicles, autophagosomes, and lysosomes (Gowrishankar et al., 2017; Maday et al., 2012; Wang et al., 2016). Axons also contain mitochondria (Saxton and Hollenbeck, 2012) and a network of smooth ER (Wu et al., 2017). Many aspects of these diverse cargos and how they navigate through the complex axonal environment are unclear.

Cryo-electron tomography (cryo-ET) is becoming an invaluable tool to understand cellular ultrastructure (Albert et al., 2020; Allegretti et al., 2020; Bykov et al., 2017; Jordan et al., 2018; Wietrzynski et al., 2020), and it can provide subnanometer-resolution structural information in situ (Himes and Zhang, 2018; von Kügelgen et al., 2020; O’Reilly et al., 2020). Previous work provided insight into neuronal MT architecture and synaptic compartments (Atherton et al., 2018; Fukuda et al., 2017; Garvalov et al., 2006; Lučić et al., 2007; Schrod et al., 2018; Tao et al., 2018a). In this study, we focus on imaging the shaft of mammalian sensory axons and provide novel observations concerning the MTs and internal compartments.

## Results

### Cryo-ET of mammalian and *Drosophila melanogaster* (Dm) neurons

Cryo-ET image quality is highly sensitive to sample thickness and limited to specimens that are less than ∼400 nm deep (Rice et al., 2018). Focused ion beam milling can thin samples, but this multistep procedure reduces the throughput for data collection (Wagner et al., 2020). We therefore directly imaged parts of axons that are sufficiently thin to be electron transparent.

**Figure 1.**
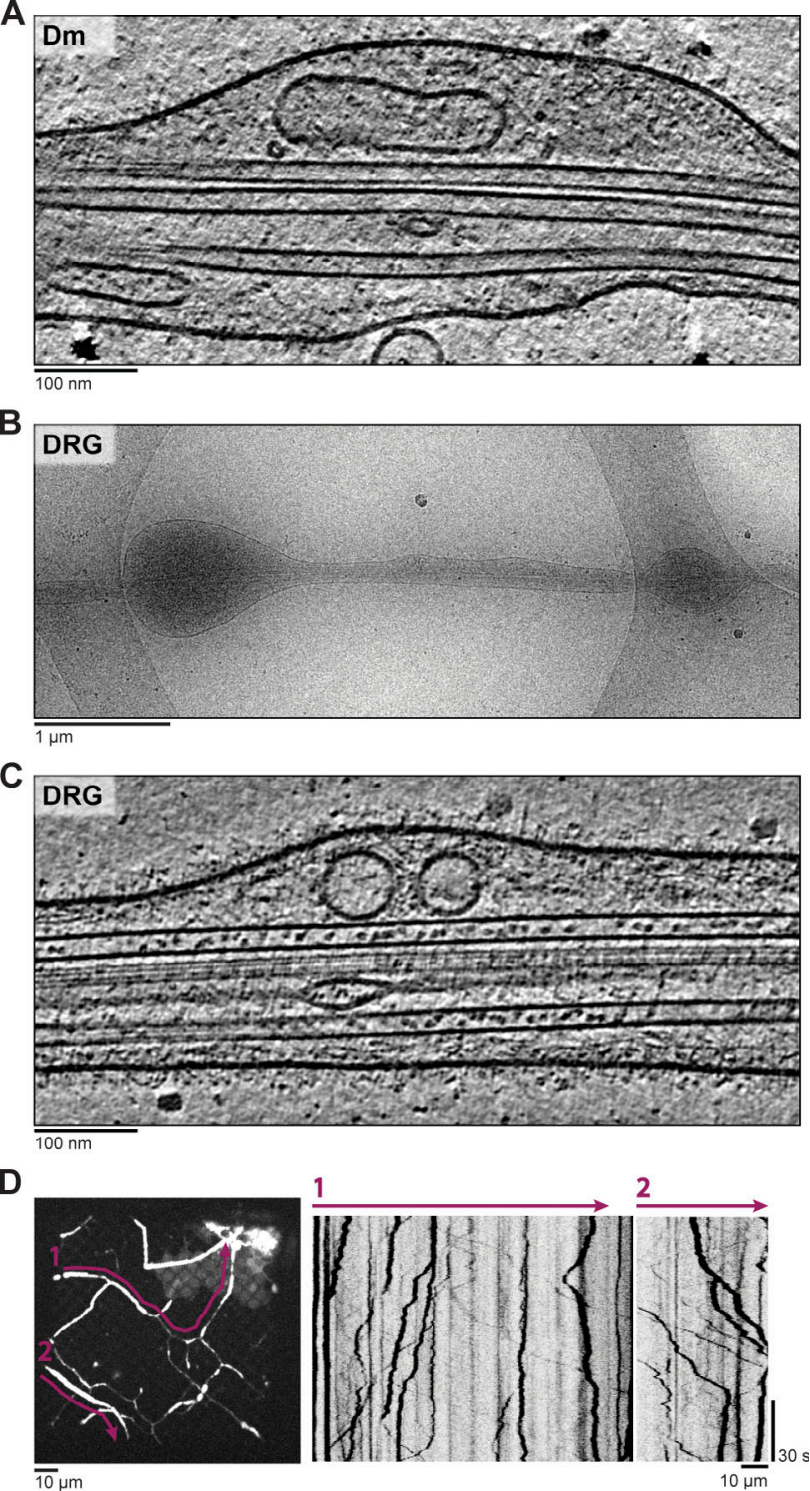
**Cryo-ET of Dm and DRG neurites.** **(A)** Tomogram slice showing MTs and organelles in a Dm neurite. **(B)** Overview of a thin DRG axon targeted for cryo-ET. **(C)** Slice of a DRG tomogram acquired at site in B. **(D)** Kymographs from a movie of DRG neurons grown on an EM grid, labeled with LysoTracker. Left panel shows maximum intensity projection of the movie. Purple lines indicate the positions used for kymographs.

We tested two strategies for neuronal culture on EM grids. We first established neuron cultures from Dm larval brains, as their neurites serve as a model system to study the cytoskeleton (Prokop, 2020) and are narrow (Egger et al., 1997). We found long neuronal processes extended over the EM grid surface within 2 d. In a cryo-ET dataset of these thin neurites, we could resolve their internal features (Fig. 1 A) but could not easily distinguish between axons and dendrites. We next isolated mammalian dorsal root ganglion (DRG) neurons, which transmit information from the periphery to the spinal cord and exclusively extend axon-like processes from their cell bodies (Kleele et al., 2014; Nascimento et al., 2018). These axons contain a uniform polarity MT array and are widely used to study MT-based axonal transport (Akin et al., 2019; Cheng et al., 2018; Delcroix et al., 2003; Gumy et al., 2017; Maday and Holzbaur, 2014). We found these cells extended axonal processes over the EM grid surface within 4 d (Fig. 1, B and C). Although DRG axons had fewer regions accessible for cryo-ET imaging than Dm neurites, they were more robust during handling and freezing. We observed that LysoTracker-labeled vesicles moved bidirectionally in the DRG neurons grown on EM grids (Fig. 1 D), indicating they are capable of axonal transport. We acquired high-quality tomograms in the thin parts of DRG neurons and focused our analysis on these data.

For this study, we acquired two datasets. The first (30 tomograms) included relatively thick regions, allowing us to survey both large and small organelles. Tomograms thicker than ∼300 nm had lower contrast and so were excluded from our analysis of the cytoskeleton. Our second dataset focused on regions thinner than ∼200 nm. All 39 tomograms were of sufficient quality to follow the paths of filaments, including MTs.

### Cytoskeletal components observed in axons

**Figure 2.**
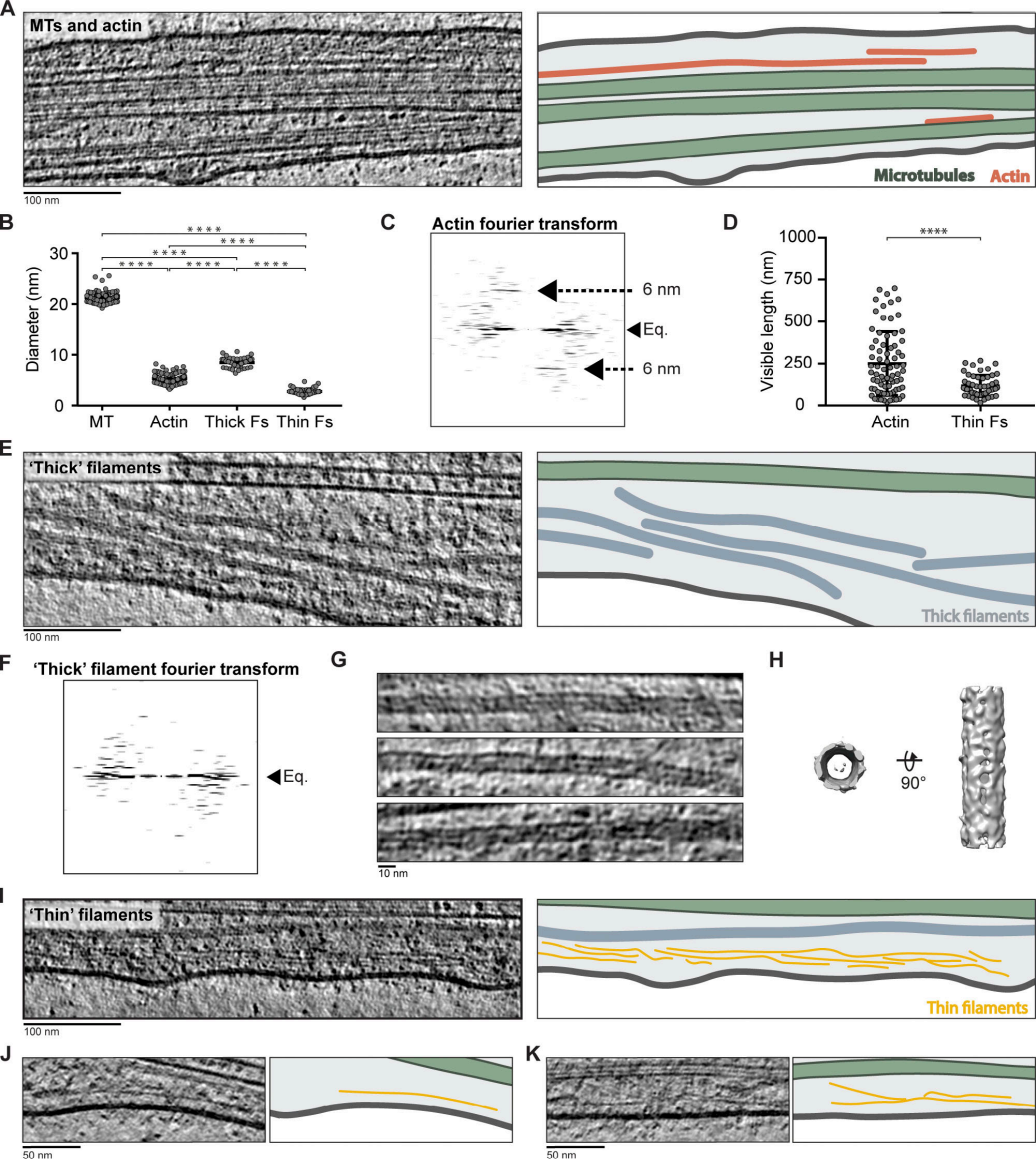
**Cytoskeletal filaments in DRG axons.** **(A)** Tomogram slice showing MTs and actin. Cartoon illustrates the plasma membrane (dark gray), MTs (green), and actin (orange). **(B)** Measured diameters of MTs (21.2 ± 1.0 nm, *n* = 113), actin (6.1 ± 1.1 nm, *n* = 91), thick filaments (thick Fs; 8.5 ± 0.8 nm, *n* = 62), and thin filaments (thin Fs; 2.8 ± 0.6 nm, *n* = 48). ****, P < 0.0001, unpaired two-tailed *t* test. Error bars show mean and SD. **(C)** Fourier transform of an actin filament. Black arrows show the position of the equator (Eq.) and layer lines. **(D)** Measured length of actin (251 ± 190 nm, *n* = 75) and thin filaments (116 ± 62 nm, *n* = 53). ****, P < 0.0001, unpaired two-tailed *t* test. Error bars show mean and SD. **(E)** Tomogram slice of thick filaments (blue in cartoon). **(F)** Fourier transform of thick filaments. **(G)** Tomogram slices showing variations in diameter of thick filaments. **(H)** Subtomogram average of thick filaments. **(I–K) **Tomogram slices showing example thin filaments (yellow in cartoons).

We observed four distinct classes of filaments in the axoplasm. The first were MTs (Fig. 2 A), which were present in all but two tomograms. They ran in parallel arrays and typically spanned the full length of the imaged volume, suggesting they are longer than our ∼1.2-µm field of view. We provide detailed analysis of these below.

The second class was actin, which we identified based on its characteristic “zig-zag” appearance (Fig. 2 A), its diameter of 6.1 ± 1.1 nm (Fig. 2 B), and the 6-nm layer lines in Fourier transforms (Fig. 2 C; Chou et al., 2019; Narita et al., 2012). We observed actin filaments in 48 out of 64 tomograms. In comparison to MTs, actin filaments were short, with an average length of 251 ± 190 nm (Fig. 2 D). Similar to previous cryo-ET studies of mammalian axons (Hoffmann et al., 2021; Lučić et al., 2007; Schrod et al., 2018), the observed actin filaments ran roughly parallel to the MT network rather than forming rings around the axon shaft. This actin may correspond to “deep” actin, which is dynamic and transported slowly through axons (Ganguly et al., 2015).

The identity of the other filament classes was less clear. One had a diameter of 8.5 ± 0.8 nm (Fig. 2 B, thick Fs) and typically ran the full length of the imaged volume (Fig. 2 E). We observed 83 of these “thick” filaments in 41 tomograms. Their diameter was similar to purified neurofilaments (Wagner et al., 2004) and nonneuronal intermediate filaments previously observed in situ (Mahamid et al., 2016). DRG neurons express intermediate filaments, including three neurofilament isoforms and peripherin (Fornaro et al., 2008). The thick filaments are therefore likely intermediate filaments.

Current models suggest intermediate filaments are built from coiled-coil dimers that oligomerize into bundles and anneal end-on-end to form long polymers (Herrmann and Aebi, 2016). The thick filaments in our tomograms showed no clear reflections in a Fourier transform (Fig. 2 F), suggesting that they are heterogenous in composition or that the repeating unit is difficult to detect. In raw images, the filaments appear to vary in diameter, and no repetitive features are visible along their length (Fig. 2 G). Their core contains discontinuous density, which is lighter than the filament walls but darker than the cytoplasm. Subtomogram averaging suggests they are tubular (Fig. 2 H).

The final class of filaments were 2.8 ± 0.6 nm in diameter and significantly shorter than the others, with an average length of 116 ± 62 nm (Fig. 2, B and D, thin Fs). These “thin” filaments have not previously been described by cryo-ET. They typically ran parallel to the MTs and sometimes appeared branched or in groups (Fig. 2, I–K).

In summary, we observed that MTs, actin, and intermediate and thin filaments form a loosely packed parallel array. Together with the ER network discussed below, these components form the environment that must be navigated by membrane-bound organelles.

### Multivariate statistical analysis (MSA) classification for MT polarity determination

Cellular MT structure has been extensively studied by resin-embedded and freeze-fracture EM (Heidemann et al., 1981; Hirokawa, 1982; Höög et al., 2011; McIntosh et al., 2018; O’Toole et al., 2003; Schnapp and Reese, 1982). More recently, higher-resolution cryo-ET has revealed the structure of MTs in vitro (Atherton et al., 2017; Guesdon et al., 2016; Schaedel et al., 2019) and described their overall appearance in nonneuronal cells (Chakraborty et al., 2020; Kiesel et al., 2020; Klena et al., 2020; Leung et al., 2021) and neurons (Atherton et al., 2018; Bouchet-Marquis et al., 2007; Fischer et al., 2018; Garvalov et al., 2006; Lučić et al., 2007; Schrod et al., 2018). Here, we performed a detailed analysis of MTs in axons to ask outstanding questions about their growth and stabilization.

**Figure 3.**
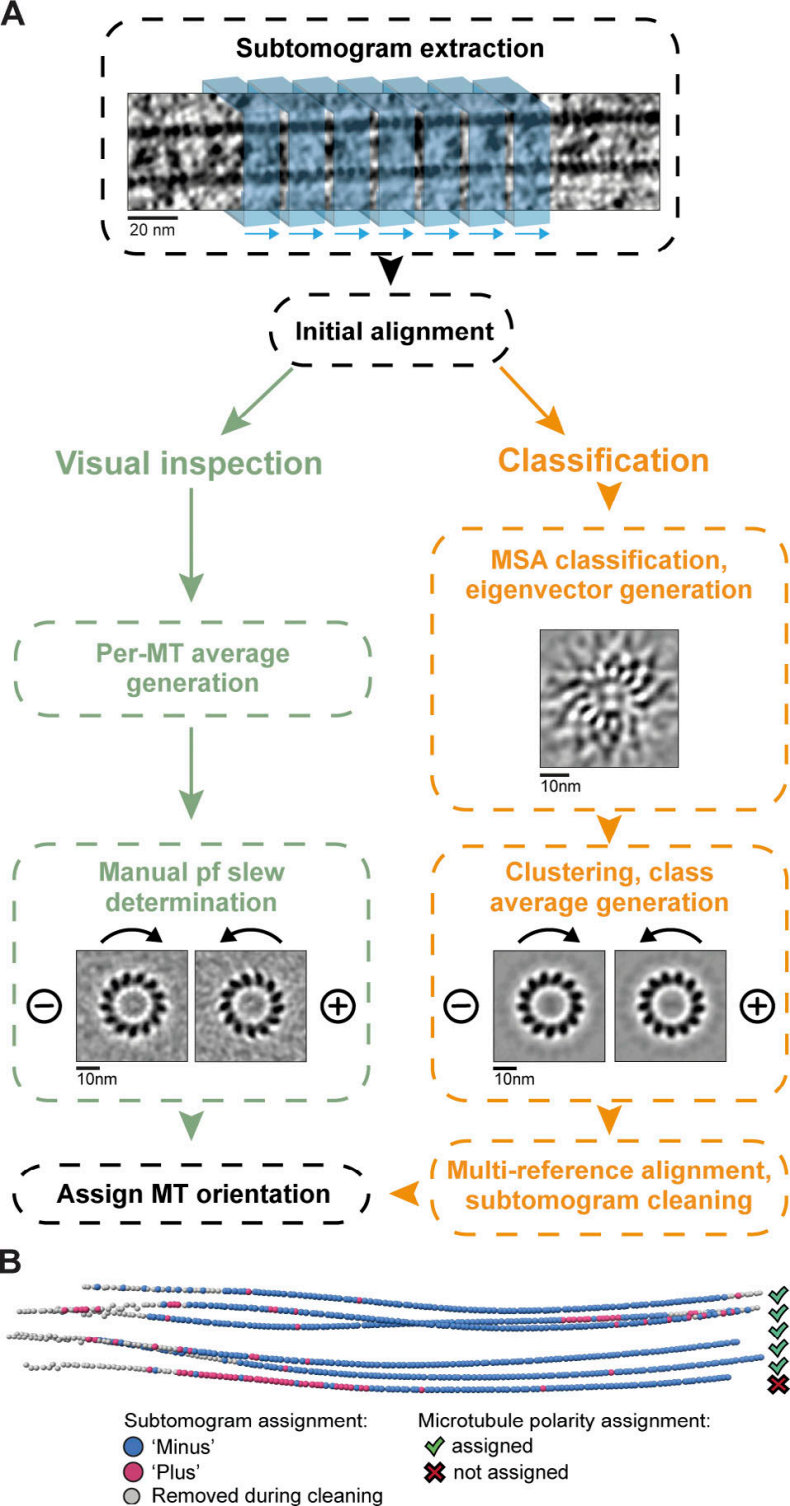
**Flow chart for MT polarity determination by visual inspection or classification.** **(A)** Subtomograms (blue boxes) were extracted along each MT then pooled and aligned. For visual inspection (green boxes), we generated subtomogram averages for each MT and used the radial slew in their projections to determine the polarity. For classification (orange boxes), we used MSA to generate eigenvectors, one of which described polarity differences. We clustered the subtomograms along this eigenvector and used the resulting averages as references in one round of MRA. We assigned the polarity of each MT based on the proportion of subtomograms in each class. **(B)** Subtomogram class distribution displayed along MTs in one tomogram. The polarity of five out of six was assigned.

Control of MT orientation ensures proper axonal polarization and transport (Witte et al., 2008). To determine whether our DRG axons contain uniformly oriented MTs, we analyzed the polarity of 271 MTs across 62 axons (a total of 75 µm long). In our initial polarity analysis, we generated low-resolution subtomogram averages of each MT (Fig. 3 A). These display a radial slew of tubulin subunits depending on the MT orientation (Bouchet-Marquis et al., 2007; Sosa and Chrétien, 1998). When projections are viewed from the minus end, the slew is in a clockwise direction, while those viewed from the plus end show an anti-clockwise rotation. We visually inspected projections of each MT average and assigned the polarity of 243 out of the 271 MTs.

To make this analysis more automatic, we implemented a subtomogram classification approach (Fig. 3 A). We first used MSA to generate ∼40 eigenvectors describing the variance in the subtomograms. We identified the eigenvector representing the difference in polarity and used it to obtain class averages corresponding to plus end– or minus end–oriented MTs. Next, we performed one round of multireference alignment (MRA), providing the plus- and minus-end MT references, and then removed subtomograms with poor cross-correlation. We assigned the MT polarity if more than 70% of subtomograms from that MT were in the same class. An example tomogram with an MT that did not reach this threshold is shown in Fig. 3 B. We ran this analysis on axons that were sufficiently thin and well aligned. The results of the classification-based analysis agreed with the visual inspection and allowed us to assign the polarity of 10 additional MTs.

Taking the combined visual and classification-based analysis, we found 245 MTs had the same polarity as all other MTs in the axon, whereas 5 ran in the opposite direction. The remaining MTs either had unclear polarity (18) or were in an axon containing only one MT (3). This agrees with low-resolution EM studies (Baas et al., 1988; Heidemann et al., 1981) and fluorescence tracking of MT ends (Kleele et al., 2014), which reported that ∼95% of MTs in mammalian axons have uniform polarity. These results show that our DRG axons contain MTs with the expected organization and provide the basis for our further analysis of the MT architecture in cells.

### MT plus and minus ends have similar morphology in axons

MTs grow by the addition of tubulin dimers to their ends. Plus ends grow frequently, while minus ends are less dynamic (Baas and Ahmad, 1992). In nonneuronal cells, growing plus ends display gently curving, flared protofilaments (pfs), although a minor proportion appear blunt, sheet-like, or curled (Höög et al., 2011; Koning et al., 2008; McIntosh et al., 2018; O’Toole et al., 2003). Similar morphologies were reported in vitro, along with more extreme architectures, including highly curled or very long sheet-like ends (Chrétien et al., 1995; Guesdon et al., 2016; Mandelkow et al., 1991). MT minus ends are inherently more stable but still require binding of stabilizing factors to prevent shrinkage (Akhmanova and Steinmetz, 2015). Minus ends in mitotic spindles are often capped by the γ-tubulin ring complex (γ-TuRC; Höög et al., 2011; Moritz et al., 2000; O’Toole et al., 2003). This factor promotes nucleation of uniformly oriented MTs in axons (Cunha-Ferreira et al., 2018; Sánchez-Huertas et al., 2016), but it is unclear if minus ends remain bound by γ-TuRCs. We therefore set out to analyze the morphology of MT ends in axons.

Across all our DRG tomograms, we found 28 MT ends and could determine the identity of 22 of them. Of these, 11 were minus ends, and 11 were plus ends.

**Figure 4.**
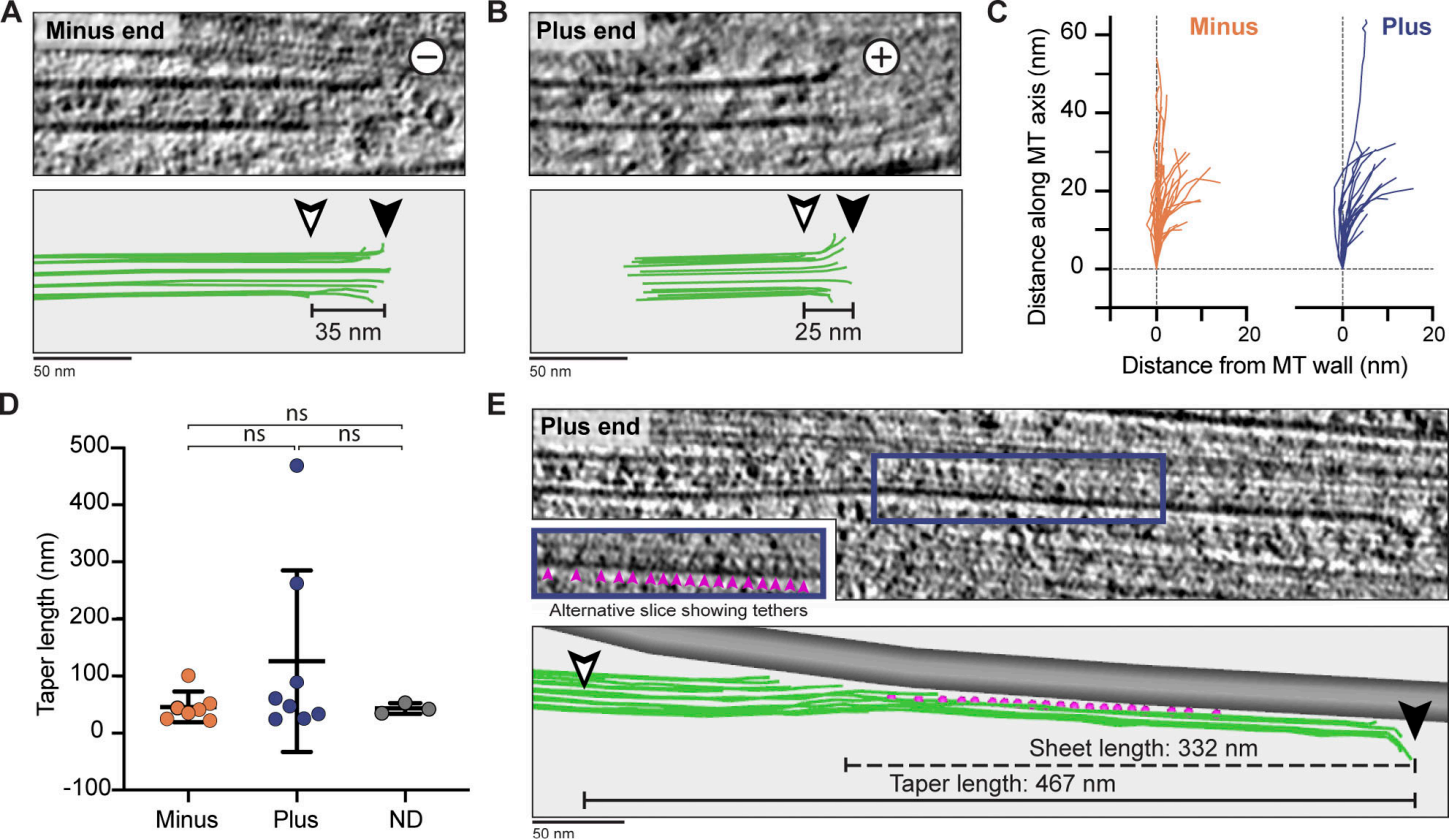
**MT minus and plus ends have similar morphology in DRG axons.** **(A)** Tomogram slice showing the morphology of an MT minus end. Bottom panel: 3D model of individual pfs (green lines) generated in IMOD. Distance between longest (black arrowhead) and shortest (white arrowhead) pf is given (taper length). **(B)** As in A, for MT plus end. **(C)** Path of individual pfs after deviation from MT wall. pfs extending from minus (orange, *n* = 49) and plus (blue, *n* = 24) ends are shown. **(D)** Taper lengths for MT ends assigned as minus (46 ± 27, *n* = 7), plus (127 ± 159, *n* = 8), or ND (43 ± 9, *n* = 3). ns, P = 0.2090 for plus versus minus, P = 0.4016 for plus versus; ND, P = 0.8668 for minus versus ND, unpaired two-tailed *t* test. Error bars show mean and SD. **(E)** Tomogram slice showing MT plus end with taper length of 467 nm. Blue box indicates region displayed inset. This uses a different tomographic slice at the same magnification to show the tethers (pink arrowheads). In the 3D model, MT pfs are green, adjacent MT are gray, and tethers are pink. Arrowheads are as in A.

**Figure S1.**
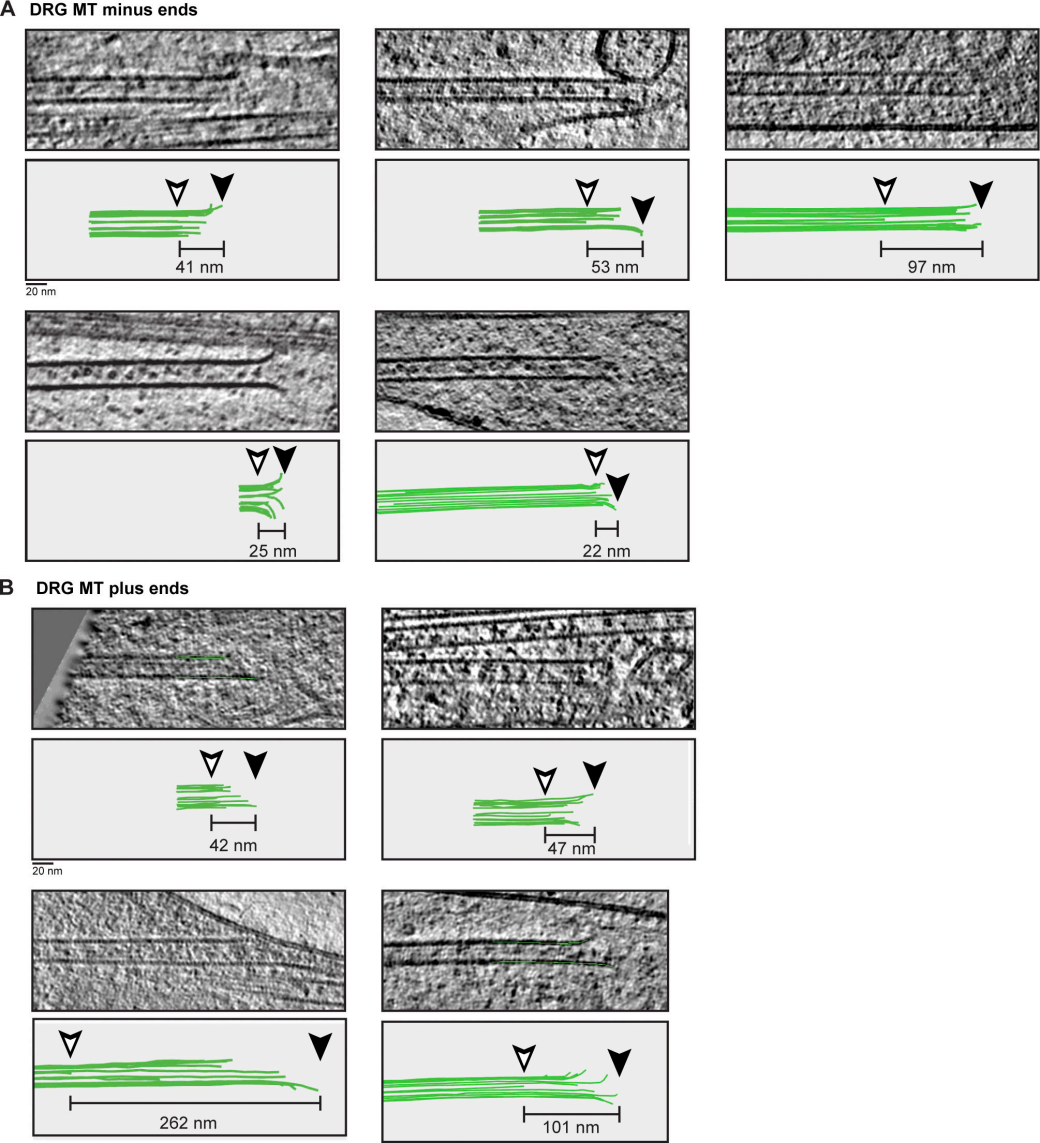
**Gallery of MT plus and minus ends from DRG axons.** **(A)** Tomogram slices and cartoons showing MT minus ends and corresponding 3D models of pfs. Taper lengths are given for each end and were calculated as distance between the longest (black arrowhead) and shortest (white arrowhead) pfs. **(B)** Tomogram slices and cartoons of MT plus ends. Annotations as in A.

Initial inspection of the minus ends showed they had straight or gently curving pfs at their ends (Figs. 4 A and S1 A). None of the minus or unassigned ends had the capped or enclosed appearance expected when γ-TuRC is bound (Höög et al., 2011; Moritz et al., 2000). Our data instead suggest that the minus ends are either “naked” or bound by smaller minus end–binding proteins such as calmodulin-regulated spectrin-associated proteins (CAMSAPs; Atherton et al., 2019). The plus ends had a similar overall appearance to the minus ends, with most having short lengths of flared pfs (Figs. 4 B and S1 B).

We traced the paths of individual pfs at a subset of ends and found a large variation in pf curvature at both plus and minus ends (Fig. 4 C). Overall, our data show that the plus and minus ends of axonal MTs have similar structure.

Besides analysis of pf curvature, MT ends can be described by measuring the distance between the longest and shortest pf (taper length). Taper lengths of up to 700 nm were seen at growing plus ends in vitro (Chrétien et al., 1995; Guesdon et al., 2016). In contrast, MT plus ends in mitotic spindles have taper lengths of 20–60 nm (Höög et al., 2011; McIntosh et al., 2018). We did not find a significant difference between the taper lengths of plus and minus ends (Fig. 4 D), with the majority being 20–60 nm. However, we found two plus ends with taper lengths greater than 200 nm, showing they are sometimes present in axons.

One of the MT plus ends with long taper length had a unique architecture. It ended in a 332-nm-long sheet (Fig. 4 E) that ran along the length of an adjacent MT and was connected to it through a series of short tethers. To our knowledge, this plus-end architecture has not been previously reported.

### DRG MTs have consistent 13 pf architecture

In vitro–polymerized MTs typically contain 11–15 pfs, while those in mammalian neurons exclusively have 13 pfs (Pierson et al., 1978; Scheele et al., 1982). Other mammalian cells also predominantly contain 13-pf MTs (Unger et al., 1990), but a recent cryo-ET study found ∼8% of MTs in HeLa cells have 12 pfs (Watanabe et al., 2020). Tubulin can be removed and replaced along MTs (Aumeier et al., 2016; Schaedel et al., 2015; Vemu et al., 2018), and the resulting lattice disruptions can affect pf number (Schaedel et al., 2019).

**Figure 5.**
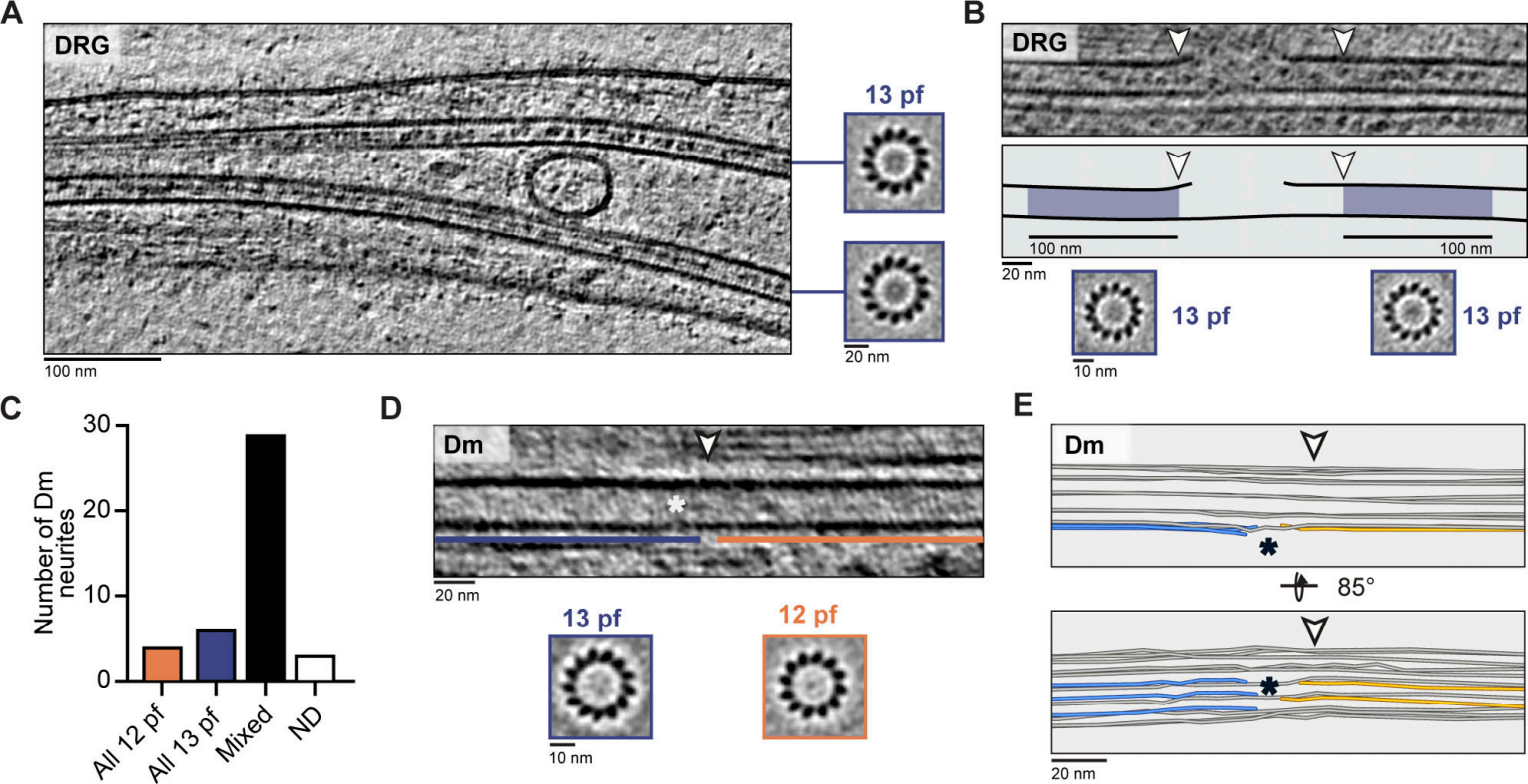
**DRG, but not Dm MTs, have consistent 13-pf architecture.** **(A)** DRG tomogram slice and associated projections of individual MT subtomogram averages showing 13-pf architecture. **(B)** Tomogram slice, cartoon, and subtomogram averages showing 13-pf architecture was maintained across lattice breaks. Cartoon shows 100-nm regions either side of the break used for subtomogram averaging (blue shading). **(C)** Distribution of MT pf architectures in Dm neurites. “Mixed” was assigned when one or more MTs with different pf number were detected within the same neurite. “ND” was assigned when all MTs had consistent pf architecture but one or more were not known. **(D)** Tomogram slice showing Dm MT at site of pf number transition. Sections assigned as 13 pfs (blue line) or 12 pfs (orange line) are indicated. Arrowhead shows transition site determined by classification. Asterisk indicates region of lattice disruption. **(E)** 3D pf tracing of the region shown in D. Annotation as in D. Terminating pfs are shown in blue on 13-pf side and in orange on the 12-pf side. Continuous pfs are in gray.

**Figure S2.**
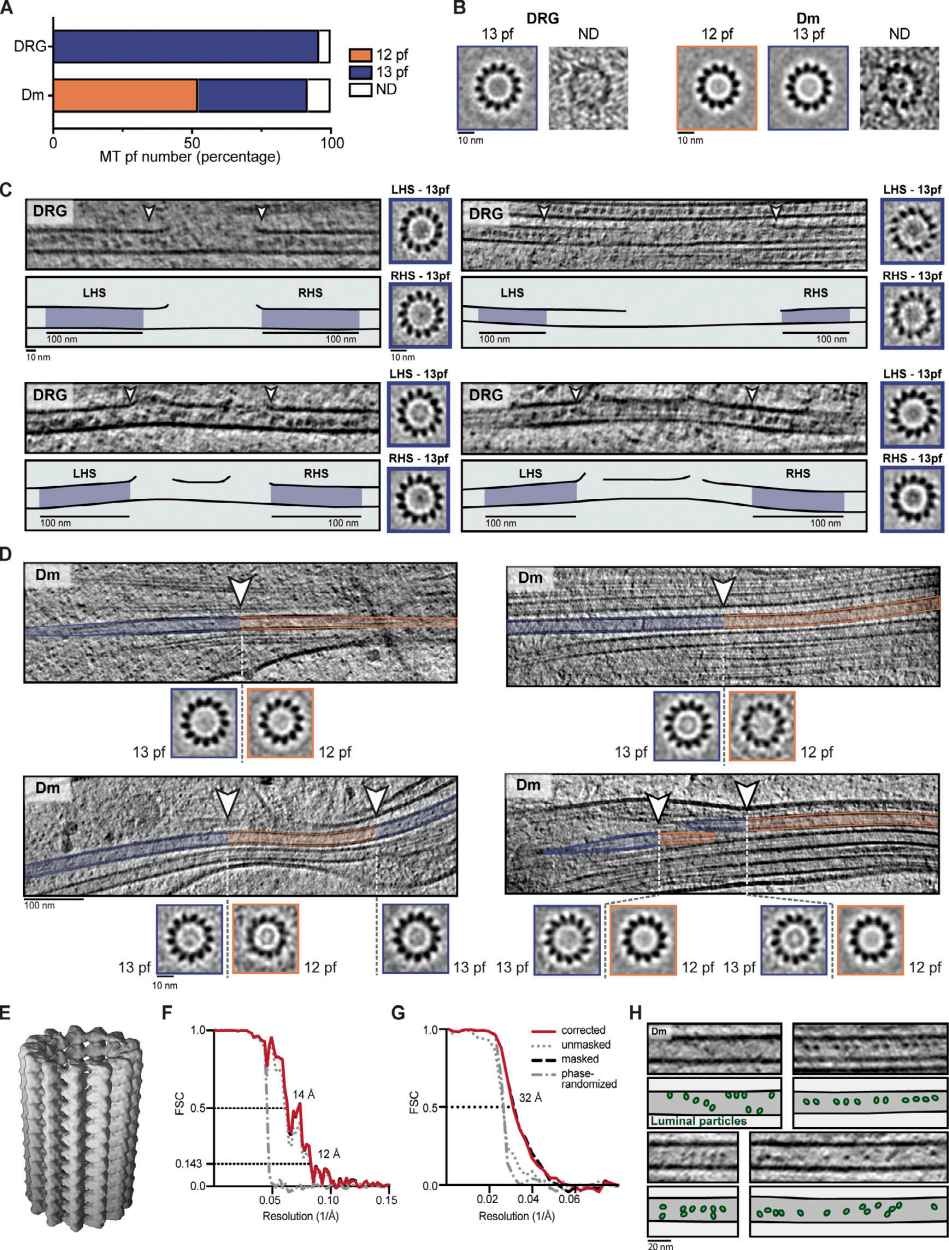
**pf number determination of MTs in DRG and Dm neurites.** **(A)** Percentage of MTs assigned as having 12 pfs, 13 pfs, or ND after visual inspection of individual MT averages from DRG (*n* = 260 13 pfs and 11 ND) or Dm (*n* = 134 12 pfs, 101 13 pfs, and 21 ND) neurites. **(B)** Examples of individual MT average projections used for assigning pf number in DRG and Dm neurites. The label above each image shows its assigned pf number. **(C)** DRG tomogram slices, cartoons, and subtomogram averages showing a 13-pf architecture is maintained at lattice breaks. Blue shading in cartoons indicate 100-nm regions either side of the MT lattice break used for subtomogram averaging. White arrowheads indicate start and end of break site. Averages for left hand side (LHS) and right hand side (RHS) of break site are shown. **(D)** Dm tomogram slices highlighting MTs that contain a pf-number transition. Regions of 13-pf architecture are shaded blue, 12-pf regions are shaded orange, and the transition site is indicated with white arrowheads. Projections of subtomogram averages from the 12-pf and 13-pf regions in each MT are shown in the bottom panels. **(E)** Surface representation of DRG MT subtomogram average. **(F) **FSC curves for the MT structure shown in E. The resolution at the cutoff of 0.143 is 12 Å. Legend is given in G. **(G)** FSC curves for the MIP subtomogram average. The resolution at the cutoff of 0.5 is 32 Å. Legend for each curve is given on the right. **(H)** Tomogram slices and cartoons of Dm neurite MTs show they contain small, globular luminal particles. Luminal particles are indicated in green in the cartoon.

To ask if any non–13-pf MTs are present in our DRG axons, we generated averages of each MT and counted the number of pfs in their projection images (Fig. 5 A). We confidently determined the architecture of 260 out of 271 MTs and found all contained 13 pfs (Fig. S2, A and B).

We observed 21 lattice breaks in our data. To find out whether local changes in the pf number were present, but not detected in our previous global analysis, we determined the number of pfs in short, 100-nm sections of MTs on either side of each break site. At the 14 sites, where we could determine the pf number on both sides, the 13-pf architecture was maintained across the breaks (Figs. 5 B and S2 C).

The strict maintenance of 13-pf architecture in DRG axons is in contrast to Dm neurites. We analyzed the architecture of Dm MTs in our cryo-ET dataset and found 52% (134/256) 12-pf and 39% (101/256) 13-pf MTs (Fig. S2, A and B). By mapping the MTs back into the neurite they originated from, we found that 29 out of 42 neurites contained both 12- and 13-pf MTs (Fig. 5 C). Previous studies showed crayfish and lobster ventral nerve cord axons exclusively contain 12-pf MTs, whereas glia contain 13-pf MTs (Burton et al., 1975). In contrast, our analysis shows 12- and 13-pf MTs frequently coexist in Dm neurites.

When MTs are polymerized in vitro, changes in pf number are often observed (Chrétien et al., 1992). However, it is unclear if these transitions exist in cells. Given our observation of MTs with different pf numbers in Dm neurites, we asked if pf number transitions are present. We adapted our classification approach for the polarity determination to map the pf number along MTs. Our analysis revealed six cases of transition between 12- and 13-pf architectures (Figs. 5 D and S2 D). During our initial inspection of the MT averages, three had been assigned as 12 pfs and two as 13 pfs. The remaining MTs were ambiguous.

At the site shown in Fig. 5 D, pf tracing showed how the MT lattice is disrupted to accommodate the change in pf number (Fig. 5 E), with three pf ends on the 13-pf side and two on the 12-pf side of the lattice disruption. This analysis showed the actual transition site was within 20 nm of the position detected by classification. Overall, our classification method was able to detect differences in pf number that were not clear by visual inspection.

We repeated the classification method on the DRG MTs but did not detect any pf-number transitions. This work provides evidence of pf-number transitions in cells and suggests they are limited to specific cell types.

### MT inner proteins are retained in the lumen at breaks and ends

**Figure 6.**
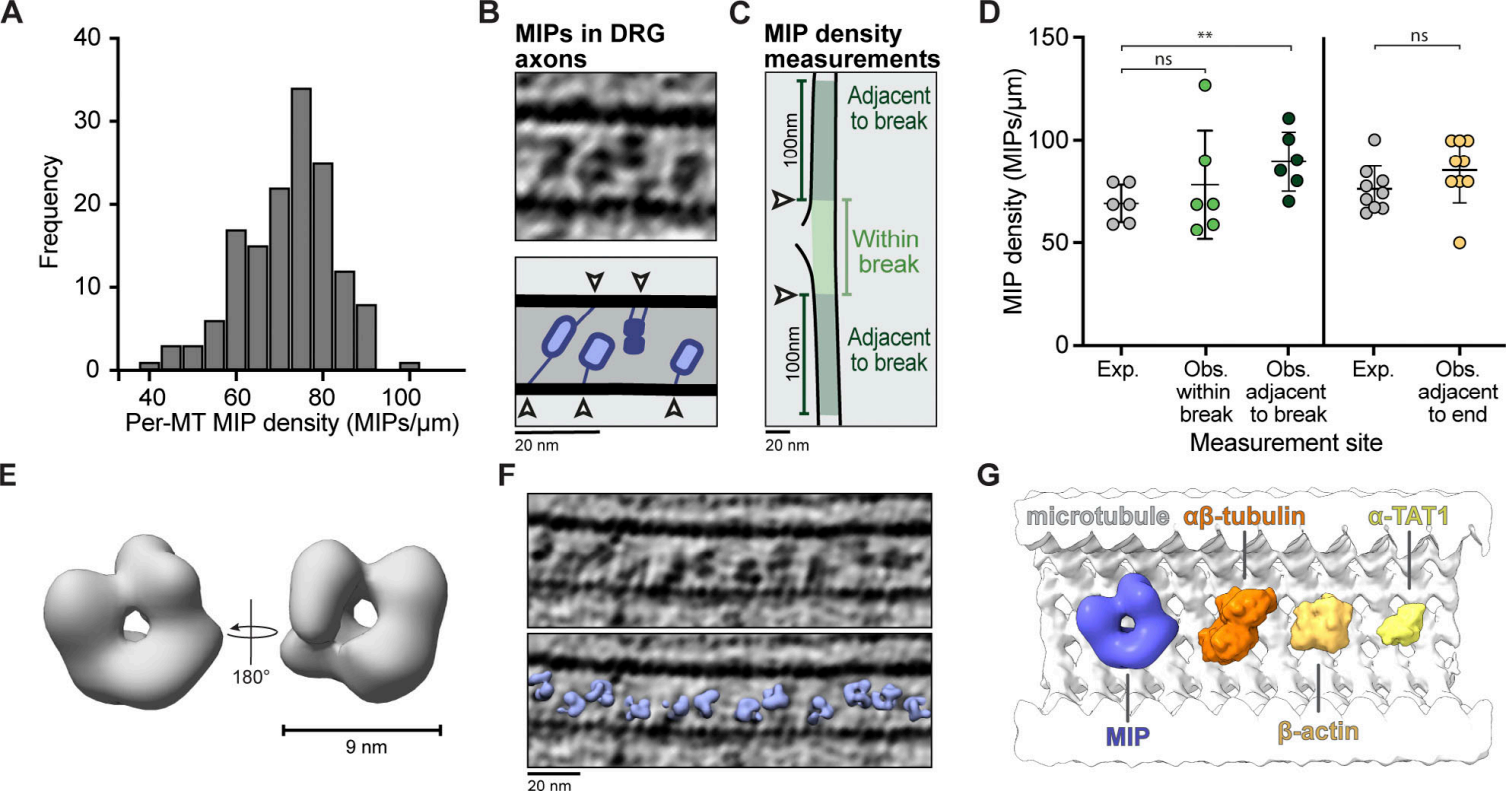
**Distribution and structure of MIPs in DRG axons.** **(A)** Distribution of MIP densities in DRG MTs (72.0 ± 10.6, *n* = 147). For each MT, the number of MIPs was divided by the MT length to give MIPs per micrometer. **(B)** Tomogram slice and cartoon showing connections (white arrowheads) between MIPs and the MT wall. **(C)** Cartoon of MT lattice break exemplifying the measurement of MIP density within (light green) or adjacent to (dark green) lattice break. Arrowheads show boundary of the break. **(D)** Comparison of expected (Exp.) and observed (Obs.) MIP density at sites of MT breaks and ends. The expected density calculated in A is given for each MT (69 ± 9, *n* = 6 for MTs with breaks, 76 ± 11, *n* = 9 for MTs with ends). The observed density was calculated within breaks (78 ± 27, *n* = 6), adjacent to breaks (89 ± 14, *n* = 6), or adjacent to ends (86 ± 16, *n* = 9). The MIP densities adjacent to breaks are the average counted either side of the break. ns, P = 0.3022 for within break; **, P = 0.0033 for adjacent to break; ns, P = 0.1025 for adjacent to end; paired two-tailed *t* test. Error bars show mean and SD. **(E)** Low-resolution subtomogram average of MIPs viewed from the front and back. **(F)** Tomogram slice showing MT and refined particle positions of MIPs after subtomogram averaging. **(G)** Size comparison of an MT (white, luminal side view), MIP (blue), α/β-tubulin dimer (PDB accession no. 6RZB, orange), β-actin (PDB accession no. 2HF3, light orange), and a-TAT1 (PDB accession no. 4GS4, yellow).

A clear feature of MTs in DRG axons are the globular particles in their lumen. These globular MIPs were identified in many eukaryotic cells and are enriched in neurons (Rodríguez Echandía et al., 1968; Burton, 1984; Garvalov et al., 2006; Bouchet-Marquis et al., 2007; Koning et al., 2008; Atherton et al., 2018; Schrod et al., 2018). Similar to a study of hippocampal neurons (Garvalov et al., 2006), we found between 40 and 100 MIPs per micron of MT (Fig. 6 A) and saw tethers between MIPs and the MT wall (Fig. 6 B). In our images, these tethers are thin, straight, and variable in length. An open question is whether the MIPs are persistently bound to the MT lattice through these tethers.

To address this, we compared the observed number of MIPs close to MT ends and lattice breaks to the expected number based on the average MIP density of that MT (Fig. 6, C and D). We hypothesized that if the MIPs were weakly associated, then they would escape from the lumen. However, we saw no significant reduction in the MIP density within breaks or close to ends and indeed a slight increase of MIPs adjacent to break sites (Fig. 6 D). This suggests MIPs are anchored to the MT, even when they are not enclosed in the lumen.

### MT inner proteins have a pore at their center

Although the globular MIPs were initially observed over 50 yr ago, their composition remains unclear. Recent work proposed that they contain MAP6 (Cuveillier et al., 2020). The AlphaFold structure prediction (Jumper et al., 2021; Tunyasuvunakool et al., 2021) of mouse MAP6 (AF-Q7TSJ2-F1) shows that it is highly disordered and lacks secondary structure apart from three short helices (Materials and methods). Given this disorder, it was surprising that many of the MIPs have a ring-like appearance in our raw images (Fig. 6 B), suggesting they have a regular structure. To test this, we analyzed them by subtomogram averaging.

To gain confidence in our processing pipeline, we first generated a subtomogram average of the DRG MTs (Fig. S2 E), which reached a resolution of 12 Å (Fig. S2 F), confirming the quality of our data. We then analyzed the structure of the MIPs. This was a challenging target for subtomogram averaging due to their small size and irregular, short distance from the MT wall. After refining the particle positions and alignment, we obtained a low-resolution (∼32 Å) subtomogram average from 3,286 particles (Figs. 6 E and S2 G) and found that the MIPs are made of multiple globular densities surrounding a central pore. Mapping the structure back into the tomograms showed the refined particle positions fit well to the MIP densities (Fig. 6 F). Connections to the MT wall are not clear in the average, indicating they are flexible. The MIPs have a diameter of ∼9 nm (Fig. 6 E), which is larger than proteins that may reside in the MT lumen such as the α-tubulin acetyltransferase 1 (α-TAT1; Coombes et al., 2016), β-actin (Paul et al., 2020), and α/β-tubulin dimers (Fig. 6 G).

Intriguingly, the Dm MTs did not contain ring-like MIPs. Instead, we saw smaller luminal densities with variable size and shape (Fig. S2 H). As MAP6 is not conserved in invertebrates (Bosc et al., 2003), the MIPs in these species likely consist of other luminal proteins. Overall, our data show that mammalian axons contain MIPs with an ordered structure and raise the question of how MAP6 could contribute to their formation.

### The ER is connected to MTs through short tethers

**Figure 7.**
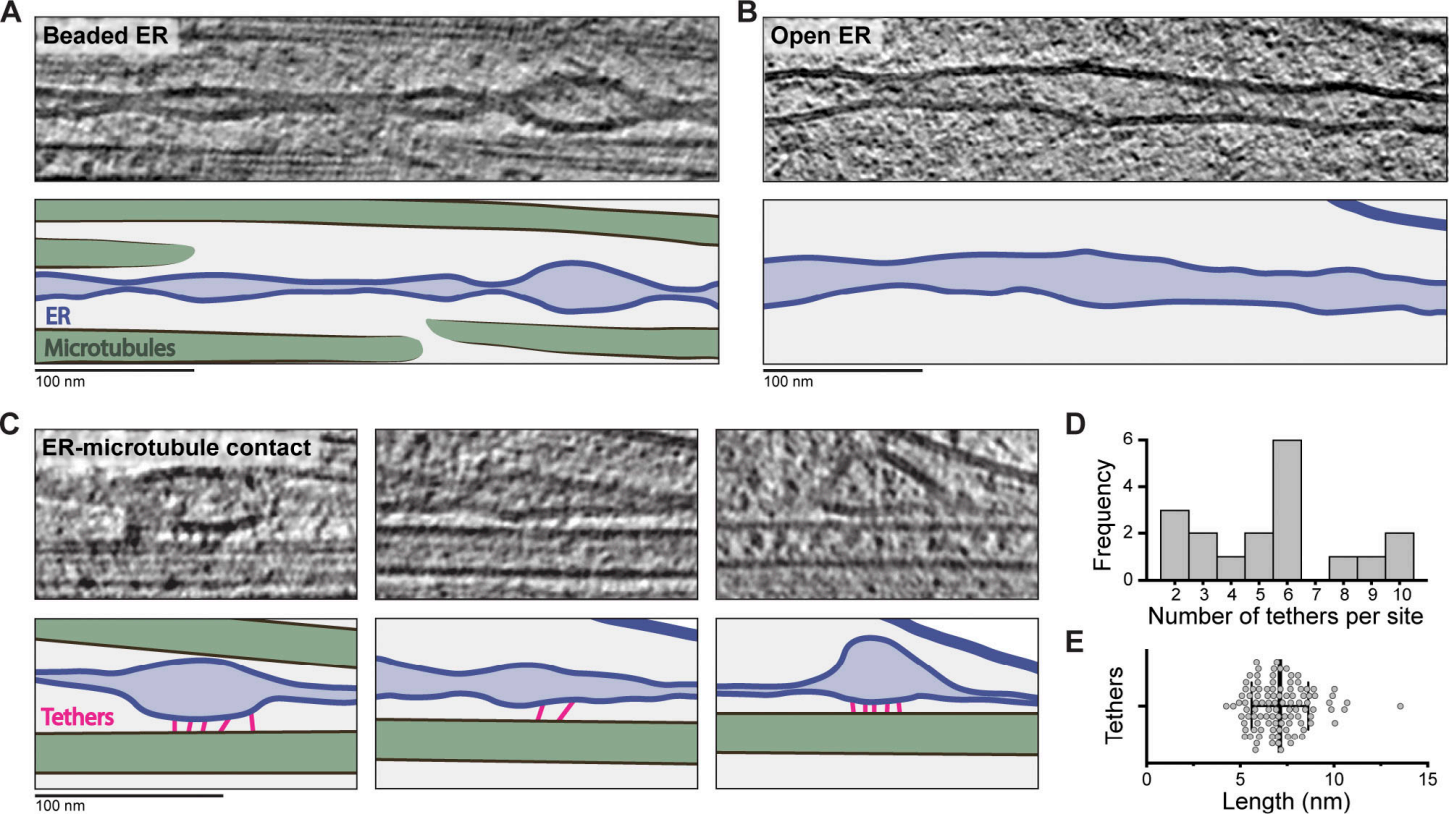
**ER–MT contact sites within axons.** **(A–C)** Tomogram slices and cartoons showing ER morphology. MTs are in green, ER membrane in blue, ER lumen in light blue, and connections between ER and MTs in pink. **(A)** Region of narrow ER with beaded morphology. **(B)** An ER section with wider lumen. **(C)** Regions with a series of short tethers linking the ER to MTs. **(D)** Frequency of tethers per site (5.5 ± 2.6, *n* = 18). **(E)** Quantification of tether length (7.1 ± 1.5, *n* = 105). Error bars show mean and SD.

Smooth ER extends throughout axons, forming a network of thin tubules (Wu et al., 2017). It is a key site for lipid biosynthesis and distribution (Vance et al., 1991), an effector of calcium homeostasis (de Juan-Sanz et al., 2017) and a source of autophagy membranes (Maday and Holzbaur, 2014). The ER in mammalian axons was previously observed by cryo-ET (Fernández-Busnadiego et al., 2015; Fischer et al., 2018; Hoffmann et al., 2021; Lučić et al., 2007; Schrod et al., 2018). Similar to these studies, the ER in DRG axons predominantly had a “beaded” appearance, where the two lipid bilayers periodically came in contact, while other stretches had open morphology (Fig. 7, A and B).

**Figure S3.**
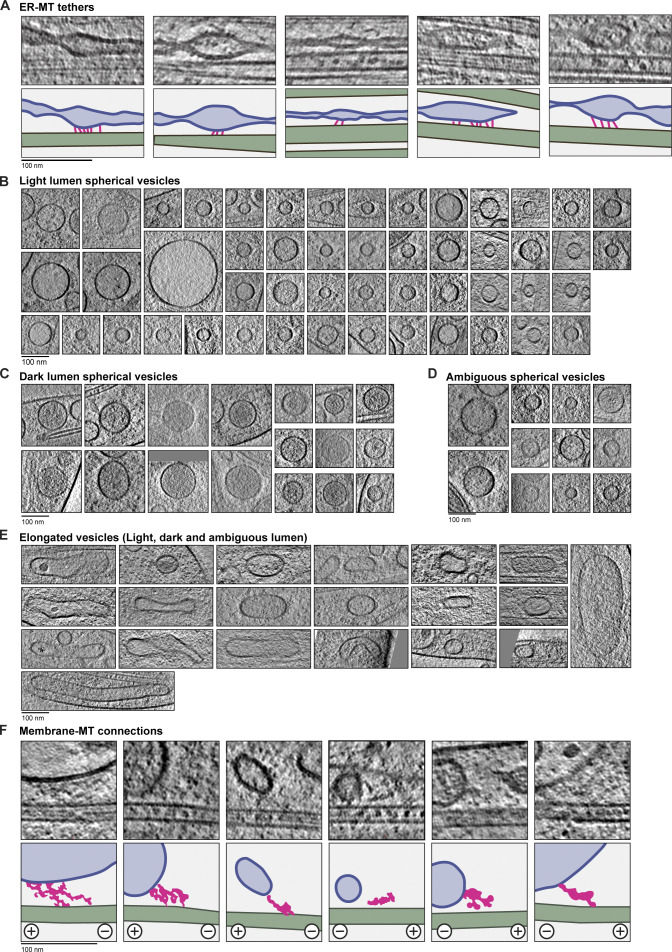
**Gallery of ER–MT tether sites and single-membrane compartments.** **(A)** Gallery of ER–MT contact sites. Bottom panels show cartoons and are colored as in A, with tethers shown in pink. **(B)** Slices through tomograms showing the morphology of light-lumen spherical vesicles. **(C)** As in B, for dark-lumen spherical vesicles. **(D)** As in B, for spherical vesicles with ambiguous lumen density. **(E)** As in B, for elongated vesicles. **(F)** Tomogram slices showing MTs close to membrane-bound compartments where connecting density is visible. In the cartoons below each example, MTs are green, membranes are blue, lumen is light blue, and connecting density is pink. The MT orientation is indicated below each example.

A key question is how the axonal ER is stretched and maintained. A previous cryo-ET study reported rare, single bridges between MTs and ER in hippocampal neurons (Schrod et al., 2018). In our data, we were able to identify clear connections between MTs and the ER (Fig. 7 C). We found 18 contact sites within 12 of 69 tomograms (Fig. S3 A). They predominantly lie in the wider regions of “beaded” ER. An average number of tethers per site was 5.5 ± 2.6 (Fig. 7 D), each being 7 ± 2 nm long (Fig. 7 E). As they are small, it is likely there are more connection sites that we were unable to detect.

### The morphology of single-membrane cargos

The axoplasm is rich in mobile membrane-bound compartments. Previous resin-embedded ET studies described the morphology and distribution of these compartments (Burette et al., 2012; Nakata et al., 1998; Sorra et al., 2006; Tao-Cheng, 2007; Wu et al., 2017; Zhai et al., 2001). Cryo-ET complemented this work to show the ultrastructure of synaptic components (Lučić et al., 2007; Schrod et al., 2018; Tao et al., 2018a). Here, we provide a comprehensive survey of the compartments present in DRG axons.

**Figure 8.**
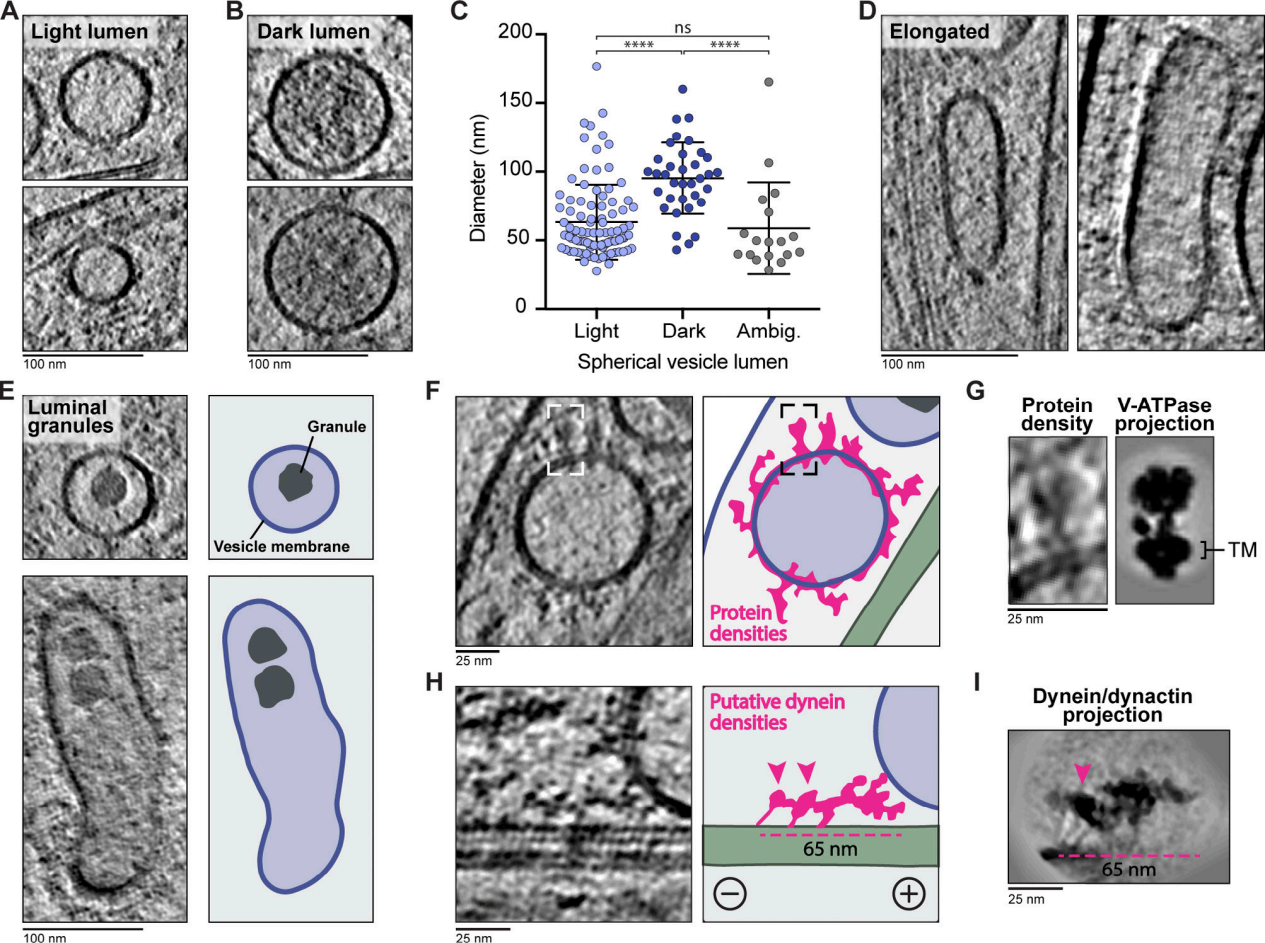
**The fine structure of unilamellar vesicles within axons.** **(A and B)** Tomogram slices of spherical light- (A) or dark-lumen vesicles (B). **(C)** Diameters of spherical light- (63.4 ± 27.4 nm, *n* = 98) and dark-lumen (95.4 ± 26.0 nm, *n* = 35) vesicles. Those with unclear lumen density are classed as ambiguous (Ambig., 59.0 ± 33.5 nm, *n* = 18). ****, P < 0.0001 for light versus dark and dark versus ambiguous; ns, P = 0.5425 for light versus ambiguous; unpaired two-tailed *t* test. Error bars shows mean and SD. **(D)** Tomogram slices of elongated vesicles. **(E)** Examples of spherical and elongated vesicles containing non–membrane-bound granules. Cartoon shows membranes (blue), vesicle lumen (light blue), and smooth sided granules (gray). **(F)** Slice through a tomogram showing large membrane-bound proteins visible on the vesicle surface. Cartoon is colored as in E, with protein density outlined in pink and MTs in green. **(G)** Enlarged view of the protein densities boxed in F, which show similarity to the projection of the low-pass filtered V-ATPase (EMD-22880). Position of transmembrane helices (TM) are indicated. **(H)** Tomogram slice and cartoon showing connections between a vesicle and MT. Coloring as in F. Arrowheads indicate globular density that may correspond to dynein motor domains, and dashed line indicates length of dynactin filament (38 nm). Plus and minus indicate MT polarity. **(I)** Low pass–filtered projection of the dynein–dynactin complex (EMD-7000) to show the possible appearance of motor complexes on MTs.

In the dataset containing both thin and thick axonal regions, we identified 247 membrane-bound compartments across 30 µm of axon length. 202 of these were single-membrane vesicles, whereas 45 contained more than one lipid bilayer. The single-membrane vesicles were either spherical or elongated. We observed 136 had a light lumen and 40 had a dark lumen, and the remainder appeared ambiguous (Fig. 8, A–D; and Fig. S3, B–D).

The majority of spherical light-lumen vesicles we observed had a diameter of 40–60 nm (Fig. 8 C), similar to synaptic vesicles (Tao et al., 2018a). The larger light-lumen vesicles (Fig. 8 C) may correspond to other axonal compartments such as synaptic vesicle precursors or early endosomes (De Pace et al., 2020; Schrod et al., 2018; Wang et al., 2016). We found that the dark-lumen vesicles were significantly larger than light-lumen vesicles (Fig. 8 C). The majority had diameters of 70–120 nm and are likely dense core vesicles (Sorra et al., 2006; Tao et al., 2018b; Zhai et al., 2001), which carry neuropeptides or neurotrophins (Bost et al., 2017; Xia et al., 2009).

A subset (47/202) of the unilamellar compartments were elongated (Figs. 8 D and S3 E). These were adjacent to spherical vesicles, suggesting they were not flattened by external force. Recent cryo-ET studies did not find an enrichment of these compartments (Schrod et al., 2018; Tao et al., 2018a), but similar structures were observed in resin-embedded EM (Hirokawa et al., 1991; Tsukita and Ishikawa, 1980; Wu et al., 2017), and they likely correspond to endosomal compartments.

In rare cases (6/202), the vesicles contained granules in their lumen (Fig. 8 E). These granules had smooth sides and were not surrounded by a lipid bilayer, suggesting they are biomolecular condensates. We saw some granules in close proximity to each other (Fig. 8 E, bottom panel), suggesting they do not readily coalesce and have gel-like rather than liquid-like properties (Alberti et al., 2019).

### Protein decorations on membrane-bound organelles

Current cellular cryo-ET methods can identify large macromolecules such as ribosomes and proteins organized in arrays (Bykov et al., 2017; Mahamid et al., 2016; Wietrzynski et al., 2020). However, identifying smaller, individual protein complexes is challenging due to the crowded nature of the cytoplasm and the low contrast in cryo-ET images caused by specimen thickness. In our thinnest, best aligned tomograms, we observed individual densities protruding from the vesicle surface (Fig. 8 F).

Although many are not easily identifiable, we speculate that a subset correspond to projections of V-ATPases (Fig. 8 G; Abbas et al., 2020). These ATP-driven proton pumps are known to reside on the surface of synaptic vesicles, endosomes, and lysosomes to control luminal pH (Ohkuma et al., 1982; Overly et al., 1995; Takamori et al., 2006).

Vesicles are transported through the axon by MT motors. Minus end–directed cargos are carried by dynein–dynactin complexes, which are 65 nm long and 30 nm wide (Urnavicius et al., 2015). As these complexes are similar in size to ribosomes and contain a 38-nm actin-like filament, we set out to determine if we could find them in our tomograms. We surveyed regions where membrane vesicles are close to MTs and identified 67 objects connecting the membrane to MTs (Fig. S3 F). In one of our best tomograms, we saw globular densities that were linked to the vesicle surface by a 60-nm-long connection (Fig. 8 H). Overall this resembles projections of a dynein–dynactin structure (Fig. 8 I; Grotjahn et al., 2018; Urnavicius et al., 2018).

We compared the orientation of our putative dynein densities to the MTs (Fig. S3 F), finding 52% (32/67) of these connections were oriented toward the minus and 48% (35/67) toward the plus end. Actively moving dyneins are expected to point toward the minus end (Imai et al., 2015). Therefore, the plus end–oriented connections we observed may be other motors or nonmotor MT-associated complexes. It is also possible they are dyneins being pulled along on plus end–directed kinesin cargo.

### Multimembrane compartments contain granules and linear membrane sheets

**Figure 9.**
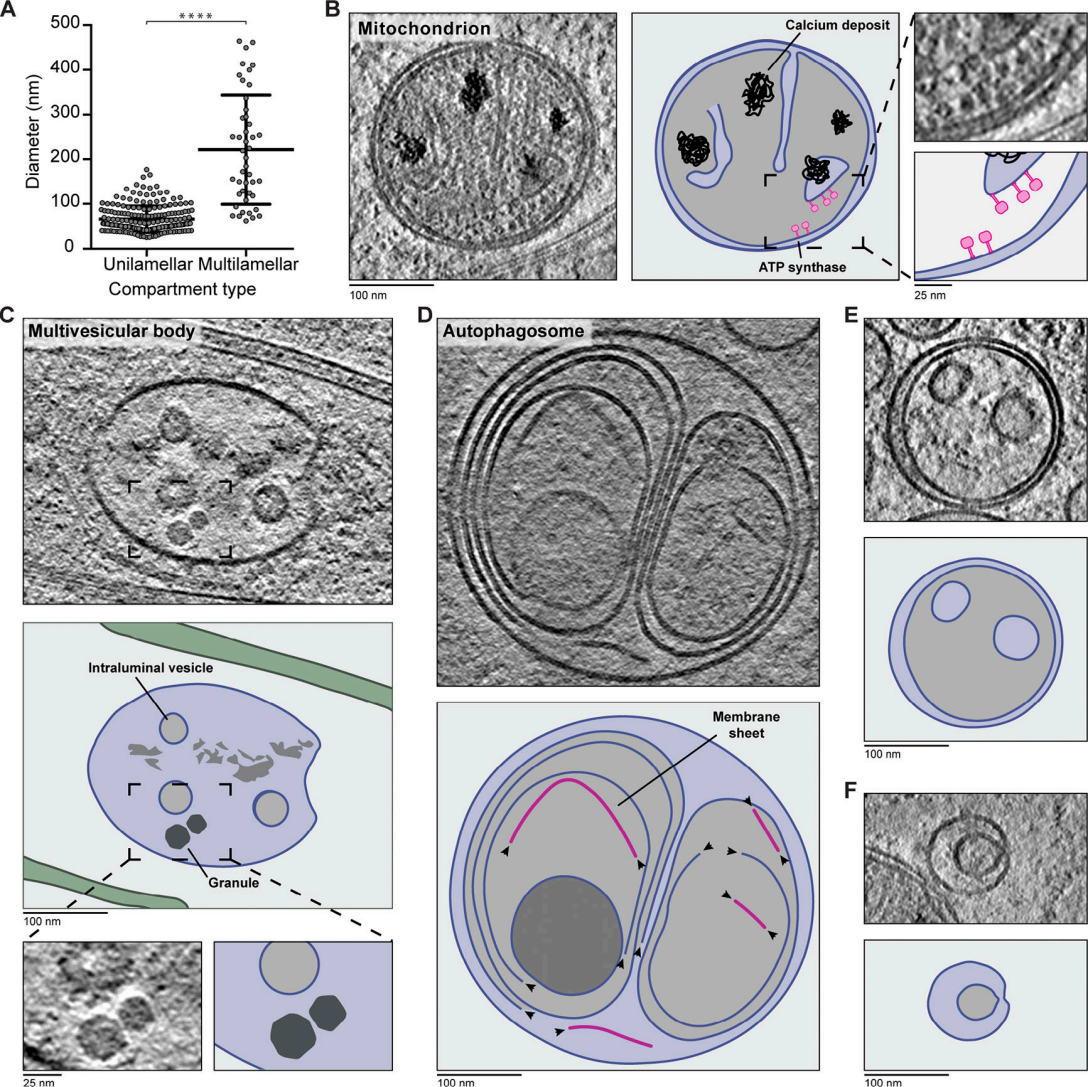
**Granules and membrane sheets within multilamellar compartments.** **(A)** Measured diameters of unilamellar (66.1 ± 29.1 nm, *n* = 202) and multimembrane vesicles (221.3 ± 122.4 nm, *n* = 45). For elongated compartments, the shortest axis is plotted. ****, P < 0.0001, unpaired two-tailed *t* test. Error bars shows mean and SD. **(B)** Tomogram slice with a mitochondrion. In the cartoon, membranes are blue, calcium deposits are black, and likely ATP synthase density is pink. Enlarged region shows ATPase heads. **(C)** Example MVB, showing ILVs and granules. The boxed area is enlarged in the bottom panel. Cartoon is colored as in B. **(D)** Tomogram slice and cartoon showing membrane sheets and ruptured vesicles within an autophagosome. Membrane sheets are pink. Arrowheads indicate exposed edges of lipid bilayer sheets. **(E and F)** Tomogram slices with multilamellar compartments of unclear identity. Cartoons are colored as in B.

The multimembrane compartments we observed were significantly larger than the single-membrane vesicles (Fig. 9 A). We categorized them into four main groups: mitochondria, multivesicular bodies (MVBs), degradative compartments, and smaller compartments with unclear identity.

**Figure S4.**
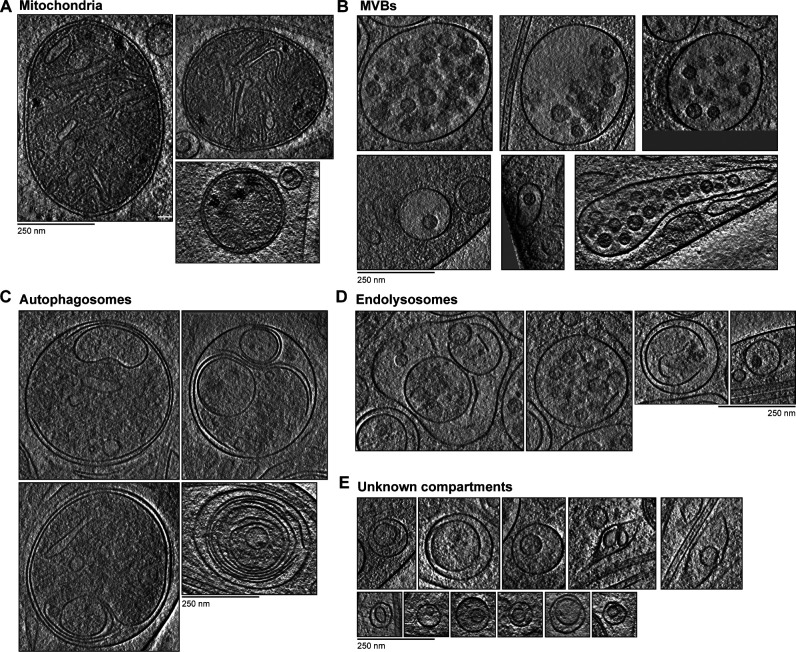
**Gallery of multilamellar compartments.** **(A) **Tomogram slices showing examples of mitochondria. **(B)** Tomogram slices for MVBs. **(C and D)** Tomogram slices showing multimembrane compartments with broken membranes in their lumen. Examples in C are more electron dense and appear more similar to autophagosomes, while those in D are less electron dense and more similar to endolysosomes. **(E)** Tomogram slices showing compartments with unclear identity.

We identified nine mitochondria (Figs. 9 B and S4 A) that resembled those previously observed in cryo-ET of axons (Fischer et al., 2018). They are double membrane compartments with a dark-lumen and electron-dense deposits previously shown to contain calcium (Wolf et al., 2017). We saw globular densities on their folded inner membrane, which likely correspond to the F_1_F_0_ ATP synthase (Davies et al., 2011).

MVBs contain numerous small, complete vesicles in their lumen (Von Bartheld and Altick, 2011; Murk et al., 2003). Our MVBs were similar to those observed in synapses (Schrod et al., 2018), but the novel feature we saw are granules in 6 out of the 10 compartments (Figs. 9 C and S4 B). These granules resemble those present in single-membrane vesicles (Fig. 8 E).

The next set of multimembrane compartments contained broken vesicles and membrane sheets (Fig. 9 D). These are likely degradative compartments such as autophagosomes or endolysosomes (Klumperman and Raposo, 2014). Some of these contained many internal membrane layers with an overall dark lumen (Fig. S4 C). Others had a more electron-transparent lumen and smaller internal vesicles but still contained broken membrane sheets (Fig. S4 D). We suspect the more dense, complex compartments correspond to autophagosomes. These are formed through encapsulation of cytoplasm including membrane-bound organelles by a double membrane and can therefore contain many membrane layers (Liou et al., 1997; Tammineni et al., 2017; Uemura et al., 2014). We assume the lighter-lumen compartments are endolysosomes, which are formed through fusion of lysosomes with mature MVBs (Luzio et al., 2000). The remaining multimembrane compartments contained large internal vesicles, which occupied much of the lumen but lacked broken membranes (Fig. 9, E and F). 17 out of 45 multimembrane compartments had this morphology, suggesting they are relatively abundant within DRG axons. It is not clear what compartments these correspond to as they have not been widely commented on in previous reports.

### Axons contain abundant protein shells that resemble virus-like capsids

**Figure 10.**
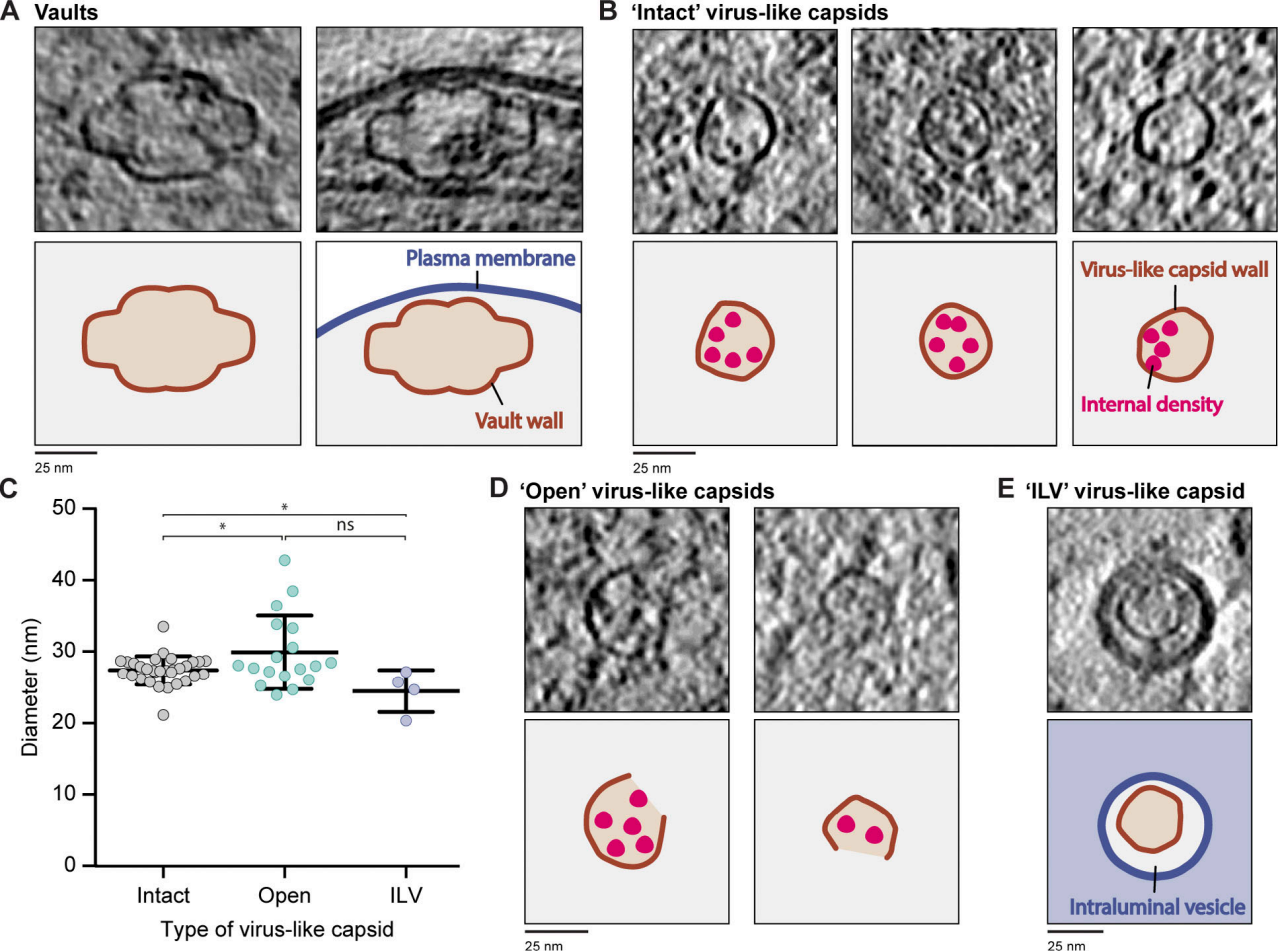
**Virus-like capsids within axons.** **(A)** Tomogram slices with axonal vaults. Cartoons depict vault protein wall in brown and membranes in blue. **(B)** Example intact virus-like capsids inside axons. Cartoons show compartment wall in brown and internal densities in pink. **(C)** Comparison of diameters from intact (27.3 ± 2.0 nm, *n* = 31), open (29.8 ± 5.1 nm, *n* = 18) virus-like capsid or those within an ILV (24.4 ± 3.0 nm, *n* = 4). *, P = 0.0186 for intact versus open, P = 0.0136 for intact versus ILV, or P = 0.0574 for open versus ILV, unpaired two-tailed *t* test. Error bars shows mean and SD. **(D)** Tomogram slices and cartoons of open virus-like capsids. Coloring as in B. **(E)** Tomogram slices and cartoons of a virus-like capsid within an ILV of an MVB. Cartoon is colored as in B, with endosome lumen in light blue and ILV lumen in gray.

**Figure S5.**
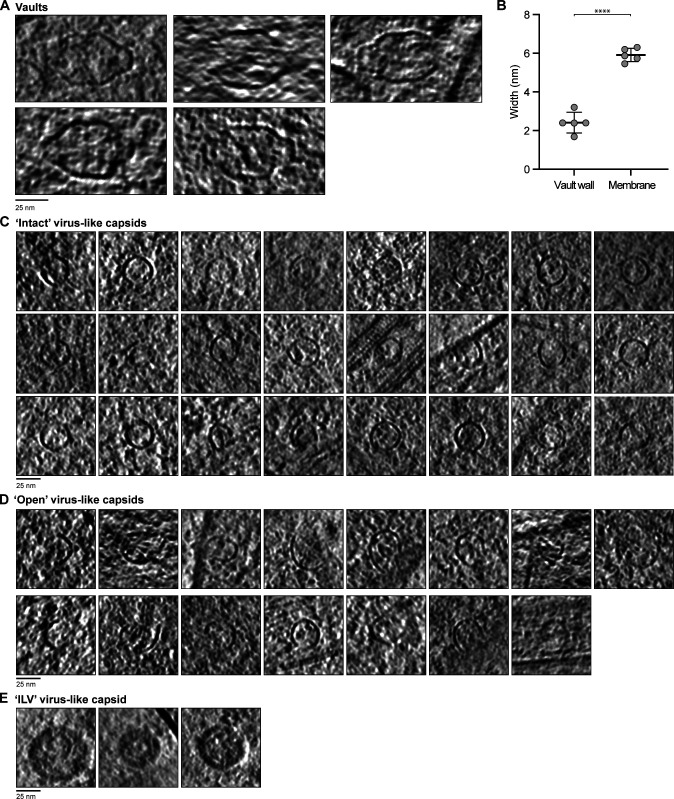
**Gallery of axonal non–membrane-bound compartments.** **(A)** Tomogram slices showing examples of vaults. **(B)** Graph showing the vault wall width (2.4 ± 0.54, *n* = 5) compared with the width of the membrane (5.9 ± 0.34, *n* = 5) within the same tomogram. Vault wall thickness was measured on three sides of each vault, with the mean and SD for each vault plotted. Membrane thickness was measured at five sites in each tomogram containing a vault, and the average for each tomogram is shown. ****, P < 0.0001, paired two-tailed *t* test. **(C–E)** Examples of virus-like capsids that are intact (C), open (D), or within ILVs (E).

In addition to membrane-bound organelles, we detected two non–membrane-bound compartments. We identified vaults based on their distinctive barrel-shaped central region which is capped on either end (Figs. 10 A and S5 A). These are highly conserved 13-MDa ribonucleoprotein complexes with elusive function (Berger et al., 2009; Kedersha and Rome, 1990; Tanaka et al., 2009). Vaults are made of a protein shell (Mikyas et al., 2004), and in our tomograms, their wall density was significantly thinner (approximately twofold) than adjacent lipid bilayers (Fig. S5 B).

The other type of non–membrane-bound compartments were smaller, with a diameter of 27.3 ± 2.0 nm (Fig. 10, B–E; and Fig. S5, C–E). Their wall thickness was similar to that of vaults, suggesting they are also made of a protein shell. We found them in 30 out of 64 tomograms, with 53 observed in total. These structures were roughly spherical with angular edges. They appeared similar to virus capsids (Goetschius et al., 2021; Vijayakrishnan et al., 2020), and so we call them virus-like capsids. Many contained discrete, globular densities in the lumen (Fig. 10, B and D), and some appeared open sided (Figs. 10 D and S5 D), suggesting they can change shape. In rare cases, they were found in the internal vesicles of MVBs (Figs. 10 E and S5 E), suggesting that they can be taken up from the cytoplasm during intraluminal vesicle (ILV) formation. On average, we found 1.4 virus-like capsids per micron of axon, showing they are unexpectedly abundant throughout axons.

## Discussion

Previous descriptions of axonal ultrastructure using cryo-ET mainly focused on synaptic regions. In this study, we characterized the axon shaft. By surveying the architecture of MTs and intracellular compartments in mammalian axons, we shed light on many aspects of MT regulation, axonal transport, and the components present in axons.

### Cytoskeletal filaments in the mammalian axoplasm

We identified MTs, actin, intermediate filaments, and previously unknown thin filaments in our tomograms. These filaments form a cytoskeletal array that runs roughly parallel to the axon. We did not observe the actin/spectrin membrane-associated periodic skeleton, which forms a sheath under the axonal plasma membrane (He et al., 2016; Xu et al., 2013). The membrane-associated periodic skeleton was also not detected in previous cryo-ET studies of mammalian neurons (Fischer et al., 2018; Hoffmann et al., 2021; Lučić et al., 2007; Schrod et al., 2018; Tao et al., 2018a). We speculate it may be excluded from the very thin regions targeted in this study.

The intermediate filaments we found have less density in their core, supporting the idea that they are tubular (Parry and Steinert, 1999; Watts et al., 2002). During the revision of this paper, two cryo-ET studies reported that the nonneuronal intermediate filaments vimentin and keratin are also tubular (Eibauer et al., 2021 *Preprint*; Weber et al., 2021). This architecture is therefore likely a common feature of these filaments.

The identity of our thin filaments is not clear. Their width is 2.8 ± 0.6 nm, which is less than half the diameter of an actin filament and twice the diameter of an α-helical coiled coil (Chou et al., 2019; Schmidt et al., 2015). Possible candidates are spectrins, which have a diameter of 1.7 nm (Ipsaro et al., 2010) and assemble into double-stranded oligomers (Machnicka et al., 2014). Spectrins can be transported in axons (Levine and Willard, 1981) and form a key component of the axonal cytoskeleton (Xu et al., 2013).

### Similar morphology of plus and minus ends in axons

We determined the polarity of MT ends in axons and found the majority of plus and minus ends have similar morphology. The minus ends were not capped, indicating they are not bound by the nucleating complex γ-TuRC and raising the question of how these MTs were generated. It is possible that the MTs were nucleated from γ-TuRCs and then released (Ahmad et al., 1994; Keating et al., 1997) or generated by another method such as MT severing (Vemu et al., 2018).

Minus ends are known to be stable (Baas and Black, 1990), but what proportion of plus ends are also stable is not clear. We detected 11 plus ends across 75 µm of axon, corresponding to 0.15 per micron length. As described in Materials and methods, this is approximately seven times the frequency seen in fluorescent imaging of the dynamic plus end–binding protein EB3 in DRG axons (Kleele et al., 2014). In the fluorescence study, stable plus ends are unlabeled and remain uncounted suggesting that the majority of plus ends we observed are stable and not dynamic. Considering the similar morphology of the plus and minus ends, they may both be stabilized by similar mechanisms.

During our analysis, we identified a previously unreported plus-end architecture, where an extended pf sheet is connected to an adjacent MT by multiple tethers of unknown identity. This arrangement was surprising, as anchoring or branching of MTs has only been described for minus ends (Alfaro-Aco et al., 2020; Basnet et al., 2018; Sánchez-Huertas et al., 2016). The connections are too short to correspond to the kinesin motors, which can bind growing plus ends in Dm dendrites (Hirokawa, 1998; Weiner et al., 2016). Instead, they may represent an unknown protein involved in guiding dynamic plus ends toward the axon tip, or they could be anchoring this plus end. As we only observed one MT end in this conformation, it is likely a rare occurrence in cells. Overall, this observation shows a new way in which MTs can be stapled together.

### Cellular control of pf number

We found a 13-pf architecture is maintained along the MT length in DRG axons, even close to lattice breaks. In contrast, MTs in Dm neurites have 12 or 13 pfs and show rare cases of pf number transition. There are many ways in which cells control the MT architecture, including MT end– (Maurer et al., 2012; Moritz et al., 2000) or lattice-binding proteins (Bechstedt and Brouhard, 2012; Moores et al., 2006; Watanabe et al., 2020), tubulin isoforms, and posttranslational modifications (Cueva et al., 2012; Savage et al., 1989). Our data suggest that DRG axons contain components to strictly maintain 13 pfs. Dm neurons may either lack these factors or contain additional regulators to promote formation of both 12- and 13-pf MTs.

### The composition of globular MIPs

The luminal particles within axonal MTs are distinct from the filamentous MIPs that line the internal walls of ciliary MTs to provide strength and stability (Ishikawa and Marshall, 2017; Nicastro et al., 2006, Sui and Downing, 2006). The globular MIPs we observe likely contain MAP6, since a mouse knockout of this component reduced the proportion of neuronal MTs containing MIPs (Cuveillier et al., 2020). MAP6 is predicted to be disordered. If it is indeed the main component of MIPs, then our subtomogram average raises the question of how it can form ordered, ring-like structures.

When tubulin was copolymerized with MAP6 in vitro, intraluminal particles were observed (Cuveillier et al., 2020), but these did not exactly resemble the MIPs in DRG axons. We therefore speculate that MAP6 brings together other intraluminal proteins to form MIP structures. Cellular MTs are known to contain α-TAT1 (Coombes et al., 2016; Szyk et al., 2014) and actin (Paul et al., 2020). Based on the comparison of their size (Fig. 6 G), either could fit into our MIP structure, whereas the α/β-tubulin dimer appears too large.

A possible function of the MIPs is to promote MT stability. Indeed, MAP6 confers MT cold stability in cells (Delphin et al., 2012; Guillaud et al., 1998). This stabilization may be through direct interactions with the MT wall (Cuveillier et al., 2020). Alternatively, the MIPs could recruit factors that contribute to MT longevity. Luminal acetylation stabilizes MTs (Portran et al., 2017). If α-TAT1 is part of the MIPs, then they could promote posttranslational modification. Based on the MIP size and tethers to the MT wall (Fig. 6 B; Garvalov et al., 2006), we expect they would prevent other components from moving freely within the lumen.

### ER tether identity

We found the smooth ER attached to adjacent MTs via thin, ∼7-nm-long tethers. These unlikely correspond to MT motors, since the kinesin responsible for ER distribution in axons contains a ∼50 nm coiled coil (Farías et al., 2019; Hirokawa, 1998) and dynein–dynactin complexes are large, 3.4-MDa assemblies (Urnavicius et al., 2018).

In addition to motors, a number of proteins are known to tether the ER to MTs in axons (Öztürk et al., 2020). These include p180 (Farías et al., 2019; Ogawa-Goto et al., 2007), P600 (Shim et al., 2008), Rab10 (English and Voeltz, 2013), REEP1 (Park et al., 2010), and Sec61β (Zhu et al., 2018). We analyzed the predicted structure of these candidates using the AlphaFold database (Jumper et al., 2021; Tunyasuvunakool et al., 2021). None clearly contained structural domains with similar dimensions to the densities we observed, suggesting the tethers are made of an as-yet-unidentified factor. The prevalence of the connections suggests they play a role in stabilizing the ER and maintaining its structure.

### Novel features of membranous compartments

Our survey of membrane-bound compartments revealed novel features of previously well-characterized organelles.

We detected granules in the lumen of both uni- and multilamellar vesicles (Figs. 8 E, S3 E, and 9 C). It is not clear what these granules are made of, but they may be a gel-like storage matrix for neurotransmitters. These have not been directly visualized, but there is indirect evidence they exist based on the rate of transmitter release from synaptic vesicles (Rahamimoff and Fernandez, 1997; Reigada et al., 2003).

Another novel feature are the unsupported lipid bilayer sheets within multimembrane compartments. Previous work showed the degradative capacity of lysosomes within axons is higher close to the cell body (Cheng et al., 2018; Yap et al., 2018). Our tomograms were collected in thin, distal axons, but we still observed broken vesicles and membrane sheets. Sheet-like membranes have also recently been observed within lamellar bodies (Klein et al., 2021) and lysosomes in the neuronal cell body (Riera-Tur et al., 2019 *Preprint*). Our data suggest that preliminary membrane breakdown occurs more widely in the axon. Alternatively, sheet-like membranes may be more common than previously appreciated.

### Virus-like capsid identity

The abundant virus-like capsids we found are similar in size to picornaviruses (13–40 nm; McFerran et al., 1971) or parvoviruses (20–25 nm; Cossart et al., 1975). However, EM images of infectious, genome-containing viruses appear “filled” (Vijayakrishnan et al., 2020), whereas our virus-like capsids do not contain internal density.

Instead of viral contamination, these nonmembranous compartments may be examples of endogenous capsid assemblies such as Arc. These transfer mRNA between neurons (Ashley et al., 2018; Pastuzyn et al., 2018), are key regulators of synaptic plasticity (Plath et al., 2006), and regulate inflammation in DRG neurons (de la Peña et al., 2021). However, purified recombinant rat Arc capsids are 32 nm in diameter (Pastuzyn et al., 2018), and Dm Arc capsids have a diameter of 37 nm (Erlendsson et al., 2020), which are both larger than the 27-nm-wide particles we observe.

Arc evolved from the Ty3/gypsy family of retrotransposons, which also form virus capsid-like structures (Dodonova et al., 2017). Related endogenous viral elements are abundant in the genomes of organisms, including yeast, flies, and humans (Abrusán et al., 2013; Feschotte and Gilbert, 2012), and can be coopted for host cell function (Frank and Feschotte, 2017).

The particles we observe could therefore either be Arc capsids displaying a reduced diameter or other, currently unknown endogenous virus-like particles.

### Outlook

A key limitation of cryo-ET is sample thickness. We show that when sufficiently thin regions are imaged, fine structures of the axon such as connections between membranes and MTs are visible. Our analysis allowed us to describe many unanticipated features within the mammalian axoplasm. These include clusters of connections between the ER and MTs, an abundance of proteinaceous compartments, and a defined structure of globular MIPs. Our work demonstrates that new features can be uncovered using cryo-ET even in a well-studied system. It also presents the challenge of identifying the components that make up these novel structures. Overall our observations demonstrate the power and limitations of cryo-ET and provide insight into the molecular architecture of the axoplasm.

## Materials and methods

### DRG neuron culture on EM grids

Primary DRG neuron cultures were derived from 6–8-wk-old mice as described in Tolkovsky and Brelstaff (2018). All procedures were performed in accordance with UK Home Office regulations and licensed under the UK Animals (Scientific Procedures) Act of 1986 following local ethical approval. Spines from male and female, wild-type (dataset 2), or transgenic GFP-RFP-LC3 (dataset 1) mice (gift from David Rubinsztein, Cambridge Institute for Medical Research, Cambridge, UK) were obtained. Cervical and thoracic ganglia were isolated from bisected spines and submerged in ice-cold HBSS (Thermo Fisher) supplemented with 20 mM Hepes, pH 7.4 (Sigma-Aldrich; HBSS+H). Ganglia from two mice were pooled and the excess connecting axon removed. After transfer into a 15-ml Falcon tube, the ganglia were washed two times in 2 ml HBSS+H and then resuspended in 1 ml HBSS warmed to 37°C and supplemented with 15 µl 20 mg/ml collagenase type IV. After incubation for 1 h at 37°C, 5% CO_2_, 1 ml warm HBSS supplemented with 100 2.5% Trypsin was added. After 15 min, the enzymatic digestion was quenched by adding 5 ml warm plating media (neurobasal media supplemented with 1 x B-27, 2 mM L-glutamine), 20 mM Hepes, pH 7.4, 100 U/ml penicillin, 100 U/ml streptomycin and 5% FBS (Thermo Fisher). Cells were washed twice in 2 ml plating media and resuspended in 1 ml plating media before being sequentially triturated using a 1,000 µl then 200 µl pipette. The cell suspension was loaded onto two ice-cold 3 ml 15% BSA-DMEM cushions and the cell bodies were pelleted for 8 min at 300 relative centrifugal force (4°C). After discarding the supernatant, the DRG cell bodies were gently resuspended in 200 plating media prewarmed to 37°C.

Quantifoil R3.5/1 Au 200 mesh grids (Quantifoil Micro Tools) were coated with a thin layer of homemade continuous carbon and then cleaned for 40 s in a Nano Clean Plasma cleaner Model 1070 (Fischione) at 70% power in a 9:1 mixture of Argon and Oxygen gas. Each grid was immediately transferred into a microwell of an Ibidi µ-slide 2 well co-culture dish (Ibidi). In a laminar flow hood, 0.1 mg/ml sterile poly-L-lysine (Sigma-Aldrich) diluted in water was added and incubated for 4–6 h at 37°C. After washing twice in water, 0.01 mg/ml sterile laminin (Sigma-Aldrich) was added for overnight incubation. Immediately before plating, the laminin solution was removed and grids were washed twice in water then once in plating media.

Cells were diluted to 60,000 cells/ml, and 40 µl was added to each microwell and placed in a 37°C incubator at 5% CO_2_. After 2 h, the macrowells were topped up to 700 µl with maintenance media consisting of Neurobasal media, 1 × B-27, 2 mM L-glutamine, 20 mM Hepes, pH 7.4, 100 U/ml penicillin, 100 U/ml streptomycin, and 100 ng/µl NGF (Peprotech). The next day, half of the media was replaced with maintenance media supplemented with 40 µM UfdU (Sigma-Aldrich) to suppress proliferation of mitotic cells. Cultures were maintained for 3–7 d before fluorescent imaging or vitrification.

### Dm neuron culture on EM grids

For Dm neuron culture on EM grids, we established primary neuron cultures from third-instar larvae of wild-type (Oregon-R) flies. Fly stocks were stored at 18°C with a 12-h/12-h light/dark cycle and transferred to 25°C during active use. Flies were transferred to a new vial three times a week. For each dissection, six third-instar larvae were obtained and cleaned by briefly submerging three times in water and once in 70% ethanol. Each larvae was then placed in a 50-µl drop of PBS. Using fine forceps, each larvae was split in half and the anterior portion retained. By pushing the mouthparts into the body cavity, the organs within the thorax were released into solution. The brain was identified and the surrounding tissue carefully removed. After dissection, the brain was transferred into a 20-µl drop of cell culture medium (CCM) by gripping an axon bundle attached to the ventral nerve cord. CCM contained 10% heat inactivated FBS, 100 U/ml penicillin, 100 U/ml streptomycin, and 2 µg/ml insulin in Schneider’s solution (Thermo Fisher Scientific) and was filtered through a 0.22-µm filter just before use.

After dissection, all brains were transferred into a 1.5-ml Eppendorf tube containing 100 µl CCM. All further steps were performed in a sterile hood. The brains were washed by removing and replacing the CCM once. Next, 100 µl prewarmed (37°C) dispersion media containing HBSS, 50 µg/ml phenylthiourea, 0.5 mg/ml collagenase type IV (Thermo Fisher), 2 mg/ml dispase II (Thermo Fisher), 100 U/ml penicillin, and 100 U/ml streptomycin was added. In addition to this enzymatic dissociation, the brains were mechanically disrupted using a micropestle and then incubated for 5 min at 37°C. 200 µl CCM was then added to quench the digestion. Cells were pelleted by spinning for 1 min at 800 relative centrifugal force and resuspended in 20 µl CCM per brain before titration using a 200-µl pipette.

Quantifoil R3.5/1 200 mesh Au grids were prepared as described for DRG samples with the following modifications. Instead of poly-L-lysine and laminin treatment, grids were coated with 0.25 mg/ml Concanavalin-A (Sigma) for 2 h at 37°C. Grids were then washed twice with water before allowing them to dry. 30 µl cell suspension was added to each grid in a microwell of an Ibidi µ-slide two-well co-culture dish. After allowing to settle for 10 min, media was added to the surrounding wells. Cultures were maintained in a 27°C incubator for 2 d before vitrification.

### Fluorescent imaging of acidic vesicles in DRG neurons

Imaging was performed on neurons grown for 3 d in vitro (DIV) at 37°C in 5% CO_2_, and all media were prewarmed to 37°C before labeling or washing. For fluorescent labeling of acidic vesicles, 50 nM LysoTracker Deep Red dye (Thermo Fisher) diluted in maintenance media was added to the culture dish containing EM grids. After 5 min, the grid was transferred to a new culture chamber containing fresh culture media and inverted so that the cell-plated side was facing the objective lens of an inverted microscope. If the grid was sufficiently flat, the focus position of a grid square could be maintained during image acquisition. Images were acquired every 500 ms for 2 min (50-ms exposure for each image) on an Andor Revolution Spinning Disk inverted microscope using a far-red filter set, 40×, 1.3-NA oil objective (334 nm/pix) and Andor iXon3 camera. ImageJ (Schindelin et al., 2012) was used for projection of maximum intensity in Z (over time) and kymograph generation.

### Vitrification of DRG and Dm neurons on EM grids

DRG and Dm neurons were vitrified by manual blotting within a Vitrobot MkII (Thermo Fisher). For DRG neuron vitrification, the Vitrobot chamber was set to 37°C and 100% humidity with blotting arms disabled. 4 × 0.7 cm strips of Whatman No. 1 filter paper were folded so the final 0.5 cm was at a 90° angle to the length of the strip and kept in a warm, humidified chamber for at least 20 min before blotting. Each grid was lifted from the culture, and 4 µl fresh culture medium containing 10 nm BSA-Au (BBI Solutions) diluted 1:6 was added before loading into the Vitrobot. Grids were blotted from the back for 3–5 s using the folded section of the filter paper strips held in a forceps before plunging into liquid ethane.

Dm neurons were vitrified using the same procedure with the following modifications. The Vitrobot chamber was set to 27°C and 100% humidity. Cultures were kept at room temperature before vitrification. 4 µl of CCM was added to each grid after removing from the culture media. This was done carefully as the Dm neurites were particularly sensitive to disruption during handling.

### Cryo-ET data acquisition

Tomograms were acquired using a Titan Krios microscope (Thermo Fisher Scientific) operated at 300 kV and equipped with an energy filter operated with slit width 20 eV. SerialEM (Mastronarde, 2005) was used to acquire tilt series between ±60° in 2° increments. At each tilt angle, a movie containing 10 frames with an exposure of ∼2 e^−^/Å^2^/tilt was acquired on a K2 camera (Gatan). The total dose for each tilt series was 110–120 e^−^/Å^2^. Two DRG datasets and one Dm dataset were used in this study. DRG dataset 1 was acquired on GFP-RFP-LC3 neurons at DIV 4 on MRC-LMB Krios 3 at nominal pixel size 3.44 Å/pix using a zero-centered tilt scheme from −30° to +60° then −30° to −60° (30 out of 32 acquired tomograms were reconstructed and analyzed). DRG dataset 2 was acquired on wild-type neurons at DIV 7 on eBIC Krios 1 at nominal pixel size 2.75 Å/pixel using a dose-symmetric tilt scheme (Hagen et al., 2017; 39 out of 43 acquired tomograms were reconstructed and analyzed). The Dm dataset was acquired on MRC-LMB Krios 3 at nominal pixel size 3.44 Å/pixel using a zero-centered tilt scheme from −30° to +60° then −30 to −60° (37 out of 42 acquired tomograms were reconstructed and included in the analysis). The defocus for each tomogram was set to between −3 and −6 µm underfocus.

### Tomogram reconstruction and visualization

The raw images were gain and motion corrected using the alignframes IMOD (Kremer et al., 1996) program. During this process, per-frame dose weighting was performed, taking into account the cumulative dose during the tilt series. Tilt series with errors during data collection were not processed, and only those with images for tilts ±52° were retained. Tomogram alignment and reconstruction were performed using the ETomo interface of IMOD. Although gold fiducials were added to the samples, data collection was not limited to regions with abundant fiducials. Where fewer than five fiducials were present, patch tracking was used to align the tilt series. All tomograms were reconstructed using weighted back projection. They were then binned by four and filtered for visualization in MATLAB using the Wiener-like deconvolution filter implemented in WARP (Tegunov and Cramer, 2019; https://github.com/dtegunov/tom_deconv). The resulting bin4, deconvolved tomograms were inspected in IMOD using the slicer window and features annotated by generation of IMOD models. Cartoons were drawn based on the density shown in the 2D images and are intended to highlight the regions containing features of interest rather than providing accurate segmentation of the density.

For subtomogram averaging of MTs, MIPs, and intermediate filaments, DRG dataset 2 tomograms were contrast transfer function (CTF) corrected by phase-flip and reconstructed by weighted back projection using novaCTF (Turoňová et al., 2017). Defoci were estimated in CTFplotter (Xiong et al., 2009) and evaluated for consistency before 3D CTF correction.

### Characterization of filaments and other axonal compartments

To determine if filaments have regular structure along their length, we performed Fourier analysis of individual filaments. For this, we aligned the filaments in the xy plane of the IMOD slicer window. The position of reflections was determined in ImageJ after contrast adjustment.

Measurements of filament lengths were performed by generating IMOD models on the bin4, deconvolved tomograms (pixel size 1.100 nm/pixel or 1.376 nm/pixel). The ends of MTs and thick filaments were rarely observed within the imaged volumes and likely have lengths longer than the ∼1.2-µm field of view. Both ends of actin and thin filaments were frequently observed, and their length was extracted from the IMOD models using the imodinfo command. This measurement is referred to as visible length, as we cannot rule out that the filaments do extend for longer than this.

The diameters of filaments and intracellular compartments were measured in a similar way. IMOD points were placed at the center of intensity peaks for MT walls or compartments on either side, and the distance between them was measured. For all quantification, mean ± SD is given.

To quantify the number and lengths of ER–MT tethers, we generated IMOD models at the 18 observed sites. Each ER–MT tether was modeled as a separate contour within the same object, and each object represented an individual ER–MT contact site. The number of tethers per site was determined from the number of contours in each object. The tether length was extracted from the IMOD model.

For quantification of the difference in vault wall thickness compared with lipid bilayers, we first calculated the average membrane thickness in five tomograms containing a vault. In IMOD, the width of five lipid bilayer sections were measured in each tomogram. We then measured the wall thickness of each vault in three places. We plotted the average vault wall thickness and average membrane thickness for each tomogram in Fig. S5 B and performed a paired two-tailed *t* test to determine the significance of their difference.

For all measurements, mean ± SDs are given throughout the text and figures. Unpaired two-tailed *t* tests were performed for statistical analysis unless stated otherwise.

### Subtomogram averaging of thick filaments

For particle picking, the path of each thick filament in filtered, bin4 tomograms was modeled in IMOD. Each filament was traced by a separate contour made up of ∼10–20 points, which were placed in the approximate center of the filament as assessed from cross-section views. The IMOD models were exported into Dynamo (Castaño-Díez et al., 2012), and particles were cropped every 8 nm using the filamentWithTorsion model workflow. After generation of a cropping table in Dynamo, this was converted into a motive list (MOTL) compatible with the subTOM pipeline (as described in Leneva et al., 2021; available at https://github.com/DustinMorado/subTOM/releases/tag/v1.1.4). All the following steps were performed in subTOM: 1,853 thick-filament subtomograms (from 13 filaments) were extracted from 7 bin2 tomograms (5.5 Å/pixel). The in-plane angles were randomized, and particles were then averaged to generate an initial reference. Particles were aligned to this reference in three iterations. The final average contains 1,049 particles after distance cleaning to a minimum distance of 8 nm. The final average was low-pass filtered to 30 Å.

### Subtomogram averaging of individual MTs for visual inspection

MTs were modeled and picked as described for thick filaments with the modification that only ∼10 points were used to trace the path of each MT. Particles were cropped every 8 nm. This distance corresponds to a tubulin dimer. An initial reference was generated by averaging all particles after randomization of in-plane angles. Particles from all MTs were pooled for alignment. After several rounds of alignment, particles originating from individual MTs were averaged. These showed the pf number and direction of tubulin subunit slew. MTs picked from the plus to the minus end show clockwise slew when projected in IMOD. Those with anti-clockwise slew were picked from the minus to the plus end. Taking into account the original picking direction and slew, we determined the orientation of 243 out of 271 MTs. Cross sections of five summed slices, as displayed in the figures, were assessed for the clockwise or anti-clockwise tubulin slew (6.88 nm [dataset 1] or 5.5 nm [dataset 2] for DRG).

For averaging of short sections of MTs, such as those adjacent to lattice breaks, MT subtomogram averages were generated as above, with the following modifications. IMOD models were generated in the region 100 nm proximal to each lattice break site. The break site boundaries were defined by the shortest pf on either side of the break. After visual inspection of the resulting MT averages, the pf architecture was clear at both sides of 14 out of the 21 lattice break sites analyzed. All of these showed 13-pf architecture.

To assess the pf architecture of MTs, we counted the pf number on five summed slices of the same projections of per-MT averages as the ones used for MT polarity determination. All 260 DRG MTs, for which we could clearly determine the pf number, had 13 pfs. The residual 11 MTs had an unclear pf architecture. For Dm, we found both 12- and 13-pf MTs. To quantify the amount of Dm neurites with either only 12-pf or 13-pf MTs or mixed pf architecture (Fig. 5 C), we looked at the pf numbers of all MTs within one neurite. We assigned them as “all 12 pf” when all MTs in the neurite had clear 12-pf architecture (4), “all 13 pf” when all MTs had clear 13-pf architecture (6), and “mixed” when one or more MTs of different architecture was detected (29). This included neurites with one or more MTs of unclear architecture. ND was assigned when all MTs had consistent pf architecture but one or more were not determined (3).

### Subtomogram classification for determination of MT polarity and pf number

For MT polarity determination by classification, a subset of tomograms with good alignment quality were selected for classification and further alignment. This included 234 MTs from 57 out of the 69 tomograms (18/30 from dataset 1 and 39/39 from dataset 2). Initial particle picking and alignment were performed in subTOM as described above. Using particles from dataset 2, we performed MSA classification. We identified an eigenvector revealing differences in MT polarity by clustering on each of the top ∼20 eigenvectors individually. Clustering on this “polarity” eigenvector resulted in particle classification into groups with opposite polarity. The eigenvector contains symmetric features which were easily recognizable in classifications of different datasets. By clustering on the polarity eigenvector, we obtained plus end– and minus end–oriented averages, which we used as initial references for one round of MRA. We then performed the same MRA classification on DRG dataset 1. For both datasets, we cleaned the data by retaining 80% of subtomograms with the best cross-correlation scores in each MT. The polarity was then assigned from the ratio of subtomograms in the minus- or plus-end classes using a custom MATLAB script. MTs with more than 70% of particles in either class were assigned to that polarity. Using this method, we determined the polarity of 221 out of the 234 MTs in this subset of tomograms. Of the 221 MTs, 10 were unclear visually but clear by classification. There were no discrepancies in the assignment of MTs by visual inspection and classification.

By adding the 10 additional MTs from the classification analysis to those visually determined, we were able to assign the polarity of 253 MTs. The combined assignment of MT polarity from visual inspection and classification was used to determine the MT end identity.

We also used MRA-based classification for automatic determination of pf number of Dm MTs. We performed three rounds of MRA after initial alignment of particles for visual inspection. We provided initial references displaying 12- or 13-pf architecture and allowed the particles to switch classes in each round. Similar to the polarity determination above, we assigned the pf architecture of each MT based on the proportion of particles in each class. MTs that had over 95% of particles in either the 12- or 13-pf class were automatically assigned. For each of the remaining MTs, we averaged the particles in the 12- and 13-pf classes separately to generate per-class per-MT averages. In most cases, only one of these averages displayed clearly defined 12- or 13-pf architecture when assessed from five overlaid slices. This indicates the particles assigned to the minor class are of poor quality, so the MT was assigned as having the architecture of the dominant class. If both class averages had poor alignment quality, then they were assigned as ND. For MTs showing clear pf architecture in both classes, we analyzed the per-class particle distribution within each MT using the “Place Object” Chimera plugin (Pettersen et al., 2004; Qu et al., 2018). In six cases, the per-class MT averages showed clear pf architecture, and the particles clustered in a patch of more than 20 particles. These MTs were assigned as containing pf transition sites. Eight other MTs had long patches containing a single class, but individual pfs were not clear in both per-class averages.

We repeated this analysis on MTs in DRG axons to test if we could find any sections of 12-pf architecture. For this, we used the 12- and 13-pf initial references from our Dm MT analysis and performed three rounds of MRA. After analyzing the class proportions, per-class averages, and their distribution along the MT, we found no evidence of transition to a 12-pf architecture.

### Quantification of pf curvature at MT ends

The curvature of individual pfs at MT ends were analyzed as described in Richard McIntosh et al. (2020). Briefly, 3D models of pfs at MT ends were generated in IMOD after extraction from the full tomogram using the IMOD program mtrotlong. Each pf was traced with a single open contour. The straight segment of the MT wall was modeled with the first and second points in the contour. The curved section of the MT was more finely sampled, with points placed every ∼4 nm. After tracing, the IMOD program howflared was used to extract the coordinates of the pf path after deviation from the MT wall. These were then imported to MATLAB for figure generation. The distance between the longest and shortest pf (taper length) at the MT end was measured manually in IMOD.

To distinguish between minus and plus ends, we used the combined assignment of MT polarity by visual inspection and classification. In figure legends and throughout the text, mean ± SD is given. Unpaired two-tailed *t* tests were used to analyze the difference in pf taper lengths at minus, plus, and undetermined ends.

To compare the number of MT plus ends in our tomograms to previous fluorescence imaging (Kleele et al., 2014), we calculated the number of ends per axon area. We estimated the average width of our imaged axons to be 0.3 µm. We surveyed a 75-µm length of axon and observed 11 plus ends in our data, giving a density of 0.15 plus ends/µm, which equals 0.49 plus ends/µm^2^. This value was compared with the 0.065 dynamic plus ends/µm^2^ previously reported (Kleele et al., 2014).

### Subtomogram averaging of DRG MTs

Particles were picked and extracted from DRG dataset 2 as described for visual inspection of MT averages, using 4 nm spacing (corresponding to a tubulin monomer). All further processing was performed using subTOM. To ensure all particles had uniform polarity, we rotated particles assigned as minus end–oriented in the previous analysis by 180° around the second Euler angle (zenit). To increase the number of particles and signal-to-noise of resulting averages, 13-pf helical symmetry was applied. To do this, we applied an in-plane rotation of 27.69° and a shift of 0.92 nm along the filament axis cumulatively 12 times to each of the 36,636 raw particles. These helical parameters are known from single-particle reconstructions of 13 pf MTs in vitro. The resulting 476,268 particles were aligned in four iterations, limiting the in-plane rotation to 28° and the shift along the filament axis to 2.2 nm in each direction, to avoid generation of duplicates. A gaussian tube was used as mask for the alignment to avoid aligning to MT inner protein densities. After alignment, particles were cleaned in two steps. First, 20% of particles with the worst cross-correlation scores were removed. Then, MSA classification and hierarchical ascendent clustering were used to identify and retain the best particles in our dataset. The resulting 139,347 particles were aligned in two further iterations. Next, the aligned MOTL was rescaled to bin2 (5.5 Å/pix), and particles were split into two half-sets, with particles originating from the same MT placed in the same half-set. The two half-sets were aligned in four iterations with fine angular sampling. We then unbinned the MOTL to bin1 (2.75 Å/pix) and aligned particles in a final iteration. The resulting MOTL was cleaned by cross-correlation score after visual assessment of the cleaning threshold in Chimera and by distance. Distance cleaning was performed using a customized script to exclude particles that were closer than 3.5 nm and had less than 25° in-plane angular difference. The resulting MOTL containing 64,528 particles was averaged and a resolution of 12 Å (gold-standard 0.143 cutoff) was determined in RELION 3.2 (Scheres, 2012). The final structure was low-pass filtered to 12 Å and sharpened with a B-factor of −700 Å^2^.

### Quantification of globular MIP abundance

To determine the density of MIPs in individual MTs, we first measured the length of each MT in dataset 2 using the IMOD models. Similar to the visual analysis of per-MT averages, we used these models for subtomogram averaging in Dynamo. We extracted subtomograms from non–CTF-corrected tomograms every 2 pixels along the length of each MT. An initial average was generated from all 56,788 particles after randomization of all alignment angles. After five rounds of alignment, we cleaned the particle list by performing “cluster cleaning.” This retains the particle with the highest cross-correlation score within a cluster of particles. A cluster was defined as having at least two particles within 5 nm. Using the Place Object Chimera plugin, the false positives and negatives were manually cleaned, resulting in 11,422 particles being retained. Per-MT densities were calculated by summing up the number of MIPs in each MT and dividing by its length.

To calculate the change in MIP density at lattice breaks and ends, we first determined the MIP density per micrometer in each MT that contained either of these features (expected). We then counted the amount of MIP particles within (observed within break) or adjacent to the lattice breaks (observed adjacent to break) and ends (observed adjacent to end) within the same MT. Taking into account the length along which particles were counted (dependent on size of lattice break or 100-nm region adjacent to breaks or ends), we then determined the MIP density per micrometer. Lattice break boundaries were defined by the shortest pf on each side of the break. Areas adjacent to breaks and ends were defined as 100-nm sections starting from the shortest pf in that break or end. For expected and observed MIP densities at MT lattice breaks and ends, mean ± SDs are given. Paired two-tailed *t* tests between expected and observed densities in each MT were performed for statistical analysis.

### Generation of a subtomogram MIP average structure

Based on our MT average structure, we identified 10 CTF-corrected tomograms containing the best-quality particles. To obtain MIP particle positions, we started with the bin4 symmetry expanded and orientation-flipped MOTL used for MT averaging above. For alignment, we used a gaussian spherical mask to focus on densities within the MT lumen and generated an initial reference by manual picking of 44 particles. After three rounds of alignment, we cleaned the MOTL using “cluster cleaning,” defining a cluster as having at least six particles within 7 nm. The bin4 alignment resulted in 3,852 particle positions but at this pixel size (1.1 nm/pixel), the ∼9 nm particles were sampled by too few pixels for accurate alignment. In addition, the MT wall was only a few pixels from the edge of the MIP, making the MT density difficult to exclude from the alignment using a gaussian mask. We therefore rescaled the MOTL to bin2 and randomized the alignment angles to remove any rotational bias that may have been introduced by the adjacent MT walls at bin4. We then aligned particles to a gaussian sphere in four iterations, allowing for 360° rotation in all directions. The resulting MOTL was cleaned manually using the Place Object Chimera plugin to remove particles with poor cross-correlation scores as a result of wrong particle positions. We aligned the residual 3,286 particles in three further iterations. To estimate the resolution, MIP particles were split into two half-sets after alignment, based on the MT they originated from. The MIP structure reached a resolution of ∼32 Å (0.5 cutoff) as determined from the fourier shell correlation (FSC) calculated in RELION. The final reconstruction was low-pass filtered to 32 Å.

### Secondary structure prediction and interpretation

AlphaFold structure predictions (Jumper et al., 2021; Tunyasuvunakool et al., 2021) for Mouse MAP6 (also known as N-STOP; AF-Q7TSJ2-F1), P180 (also known as RRBP1; AF-Q99PL5-F1), Rab10 (AF-P61027-F1), REEP1 (AF-Q8BGH4-F1), and Sec61β (AF-Q9CQS8-F1) were accessed through the UniProt database (https://www.uniprot.org). p600 (also known as Ubr4; A2AN08) is 5,180 amino acids long and so was not available in the web browser of the UniProt database or AlphaFold Protein Structure Database (https://alphafold.ebi.ac.uk/) at the time of writing. Instead, we retrieved the structure prediction for sections of the highly homologous human sequence (AF-Q5T4S7-F1 to AF-Q5T4S7-F20) from the human proteome bulk download (https://alphafold.ebi.ac.uk/download). Regions with pLDDT <70 were considered disordered.

### Online supplemental material

Fig. S1 shows a gallery of MT minus and plus ends in DRG axons. Fig. S2 shows the pf number determination for DRG and Dm MTs, FSC curves for the in situ DRG MT and MIP structures, and images of the Dm MIPs. Fig. S3 contains galleries of ER–MT contact sites, unilamellar compartments, and membrane–MT connections from DRG axons. Fig. S4 shows a gallery of axonal multilamellar compartments from DRG axons. Fig. S5 shows a gallery of vault proteins and virus-like capsids found in DRG axons.

## Data Availability

Cryo-EM maps of DRG MTs and MIPs are available at the Electron Microscopy Data Bank (EMD-12639 and EMD-12640), along with representative deconvolved bin4 tomograms of DRG dataset 1 (EMD-13600, EMD-13601, and EMD-13602) and DRG dataset 2 (EMD-13599 and EMD-13598) have been deposited. Raw data and all filtered, bin4 tomograms of DRG datasets are available at the Electron Microscopy Public Image Archive (EMPIAR) with accession codes EMPIAR-10815 (dataset 1) and EMPIAR-10814 (dataset 2). Data used for quantification are provided in BioStudies (S-BSST704). EMD-13598 (dataset 2, TS_43) contains MIPs and vaults. EMD-13599 (dataset 2, TS_41) shows intermediate filaments. EMD-13600 (dataset 1, TS_14) contains MVBs and an ILV virus-like capsid. EMD-13601 (dataset 1, TS_20) shows thin filaments, beaded ER, and protein densities on the surface of vesicles. EMD-13602 (dataset 1, TS_29) contains an MT plus end, a representative MT lattice break, and a site with ER–MT tethers. All corresponding tomograms can also be found at EMPIAR with the denominators given in brackets.
